# A schizophrenia subgroup with elevated inflammation displays reduced microglia, increased peripheral immune cell and altered neurogenesis marker gene expression in the subependymal zone

**DOI:** 10.1038/s41398-021-01742-8

**Published:** 2021-12-15

**Authors:** Hayley F. North, Christin Weissleder, Janice M. Fullerton, Rachel Sager, Maree J. Webster, Cynthia Shannon Weickert

**Affiliations:** 1 Neuroscience Research Australia, Sydney, NSW Australia; 2 School of Psychiatry, Faculty of Medicine, University of New South Wales, Sydney, NSW 2052 Australia; 3 School of Medical Sciences, Faculty of Medicine, University of New South Wales, Sydney, NSW 2052 Australia; 4 Department of Neuroscience and Physiology, Upstate Medical University, Syracuse, NY USA; 5 Laboratory of Brain Research, Stanley Medical Research Institute, 9800 Medical Center Drive, Rockville, MD USA

**Keywords:** Molecular neuroscience, Schizophrenia, Bipolar disorder

## Abstract

Inflammation regulates neurogenesis, and the brains of patients with schizophrenia and bipolar disorder have reduced expression of neurogenesis markers in the subependymal zone (SEZ), the birthplace of inhibitory interneurons. Inflammation is associated with cortical interneuron deficits, but the relationship between inflammation and reduced neurogenesis in schizophrenia and bipolar disorder remains unexplored. Therefore, we investigated inflammation in the SEZ by defining those with low and high levels of inflammation using cluster analysis of *IL6*, *IL6R*, *IL1R1* and *SERPINA3* gene expression in 32 controls, 32 schizophrenia and 29 bipolar disorder cases. We then determined whether mRNAs for markers of glia, immune cells and neurogenesis varied with inflammation. A significantly greater proportion of schizophrenia (37%) and bipolar disorder cases (32%) were in high inflammation subgroups compared to controls (10%, *p* < 0.05). Across the high inflammation subgroups of psychiatric disorders, mRNAs of markers for phagocytic microglia were reduced (*P2RY12*, *P2RY13*), while mRNAs of markers for perivascular macrophages (*CD163*), pro-inflammatory macrophages (*CD64*), monocytes (*CD14*), natural killer cells (*FCGR3A*) and adhesion molecules (*ICAM1)* were increased. Specific to high inflammation schizophrenia, quiescent stem cell marker mRNA (*GFAPD*) was reduced, whereas neuronal progenitor (*ASCL1*) and immature neuron marker mRNAs (*DCX*) were decreased compared to low inflammation control and schizophrenia subgroups. Thus, a heightened state of inflammation may dampen microglial response and recruit peripheral immune cells in psychiatric disorders. The findings elucidate differential neurogenic responses to inflammation within psychiatric disorders and highlight that inflammation may impair neuronal differentiation in the SEZ in schizophrenia.

## Introduction

Schizophrenia and bipolar disorder are severe psychiatric disorders with unknown causes affecting ~1% [1] and ~3% [2] of the population, respectively. Both disorders have common characteristics, such as the presence of psychosis [3], cognitive [4] and negative symptoms [5], and overlapping genetic risk [6–8]. They also share neuropathology of compromised inhibitory interneurons [9–11] and increased inflammation in the brain and periphery [12–15]. Evidence from epidemiological [16, 17] and genetic studies [18] suggest a pathogenic role of inflammation in schizophrenia and bipolar disorder. Therefore, the brain-resident immune cells, microglia, have been investigated in psychiatric disorders with varying results [19, 20]. Microglia density is unaltered and various microglia-specific markers are often decreased across brain regions in schizophrenia [21]. In bipolar disorder, microglia density and mRNA levels of microglia markers are unchanged in the medial frontal gyrus [22]. Regardless of reduced microglia marker expression, the prefrontal cortex of people with schizophrenia has increased expression of the pro-inflammatory cytokines interleukin (IL) 6 (*IL6*), *IL1B* and CXC motif chemokine ligand 8 (*CXCL8*, also *IL8*), and a general inflammation marker serpin family A member 3 (*SERPINA3*), which is induced in response to pro-inflammatory cytokines [23, 24]. In bipolar disorder, cortical mRNA and protein levels of IL1β and IL1 receptor 1 (IL1R1) are elevated [25]. Cytokine levels are highly variable within schizophrenia and bipolar disorder groups, which is further complicated by the anti-inflammatory effects of some medicines commonly used for treatment [26, 27]. However, pro-inflammatory cytokines are elevated in drug-naive individuals with first episode psychosis [28]. To account for the heterogeneous molecular profile within psychiatric disorders, recent studies use cluster analysis to determine subgroups within schizophrenia and bipolar disorder [13, 29, 30]. Subgrouping based on inflammatory marker expression identifies 19–46% of individuals with schizophrenia and bipolar disorder to have heightened inflammation in different brain regions [13, 23, 31, 32]. The subgroup of people with schizophrenia and high inflammation demonstrates increased expression of perivascular macrophage markers in the parenchyma [33], increased expression of astrocyte markers and astrogliosis [34], reduced trophic factor expression and exaggerated deficits in inhibitory interneuron marker expression in the cortex [23].

Inflammation regulates the production of telencephalic inhibitory interneurons in the subependymal zone (SEZ, also termed subventricular zone) [35, 36]. The SEZ adjacent to the lateral ventricles is the largest source of inhibitory interneurons in the human brain [37, 38], making it particularly relevant in psychiatric diseases. Neurogenesis is implicated in the pathophysiology of schizophrenia and bipolar disorder through post-mortem studies and genetic and environmental risk factors [39–43]. Despite recent controversy [44, 45], strong evidence for the persistence of adult neurogenesis exists in the human dentate gyrus of the hippocampus [46, 47] and the SEZ [38, 48–50]. In humans, the SEZ is a four-layered structure with a monolayer of ependymal cells, a hypocellular gap, an astrocytic ribbon and a transitory layer [51]. The astrocytic ribbon harbours neural stem cells expressing glial fibrillary acidic protein delta (*GFAPD)* [52] and antigen KI-67 (*MKI67)* when activated to proliferate [53]. Transit-amplifying progenitor cells can become neuronal progenitor cells expressing achaete-scute homolog 1 (*ASCL1)* [54] and distal-less homeobox 6 antisense RNA 1 (*DLX6-AS1*) [55]. When neuronal progenitor cells become immature neurons, they express doublecortin (*DCX)* and can migrate out of the SEZ [51] to become inhibitory interneurons in various brain regions [38, 56–58].

Neural stem and neuronal progenitor cell marker mRNAs are reduced in the SEZ in schizophrenia and bipolar disorder compared to controls [50, 59]. RNA sequencing from the same study suggests that increased inflammation and macrophage infiltration may be associated with aberrant neurogenesis in schizophrenia [50]. Maternal immune activation (MIA) models of schizophrenia support that inflammation impacts neurogenesis, as offspring exposed to inflammation during gestation have decreased density of neural stem and neural progenitor cells in the adult SEZ and behavioural deficits [36]. Thus, inflammation during development or disease progression may alter neurogenesis and lead to neuropathological abnormalities in psychiatric disorders.

Inflammation differentially regulates the various cellular developmental stages of neurogenesis depending on cytokine concentrations, immune cell type and activation state (for review see [35, 60]). Cytokines are produced in the SEZ by glia, neuronal precursors and immune cells [61, 62], but cytokines can also be transported across the blood-brain barrier [63]. The unique SEZ vasculature has sections devoid of integral blood-brain barrier components, such as astrocytic end feet and pericytes, allowing greater passive diffusion of small molecules compared to other brain regions [64]. Compromised blood-brain barrier integrity is also associated with easier immune cell infiltration [65]. In fact, neural stem and neuronal progenitor cells, which express IL1R1 [66], are found in close proximity to blood vessels [64]. IL1β signalling via IL1R1 enhances neurogenesis prenatally but reduces proliferation and promotes glial instead of neuronal differentiation in adulthood [67]. In addition, chronic IL1β overexpression depletes the SEZ of *DCX*-expressing immature neurons [67]. The types of cytokines released by immune cells depend on their activation state, which can be on a continuum from M1 (pro-inflammatory) to M2 (anti-inflammatory). Pro-inflammatory immune cells and associated cytokines generally reduce neurogenesis, whereas anti-inflammatory immune cells increase cell proliferation and promote differentiation into glia rather than neurons [68–70]. Cluster of differentiation 163 (CD163) is a marker for perivascular macrophages [71], which functions as a scavenger receptor and is involved in cytokine production [72]. *CD163* and intercellular adhesion molecule 1 (*ICAM1*), which is responsible for peripheral immune cell recruitment, both have increased mRNA expression in the prefrontal cortex of the high inflammation subgroup in schizophrenia. In contrast, microglia marker mRNA expression (ionized calcium-binding adaptor molecule 1, *IBA1*) is unchanged [33]. In the SEZ, CD163^+^ macrophages are found in close proximity to neural stem and neuronal progenitor cells, and their density is increased in schizophrenia compared to controls and bipolar disorder [50]. However, we do not know if the infiltration of macrophages is linked to high cytokine expression within the neurogenic niche or if high cytokine expression and increased macrophage density are independent.

The present study used targeted anatomical dissection of the SEZ, gene expression measurements, cluster analysis and immunohistochemistry in post-mortem brains of controls and people who had schizophrenia and bipolar disorder. We analysed mRNA expression of cytokines (*IL6, IL1B, CXCL8*), cytokine receptors (*IL6R, IL1R1*) and other inflammation-associated genes (*SERPINA3, IL6ST*). We determined whether the proportion of subgroups with a heightened inflammatory state in the SEZ differs according to diagnosis. Finally, we assessed whether gene expression of markers for resident immune cells [*IBA1*, hexosaminidase subunit beta *(HEXB)*, CD68 molecule *(CD68)*, purinergic receptor P2Y12 (*P2RY12*), purinergic receptor P2Y13 (*P2RY13*)], astrocytes [vimentin (*VIM*), *pan*-*GFAP*], peripheral immune cells [*CD163, CD64, CD14*, Fc fragment of IgG receptor IIIa *(FCGR3A)*] and neurogenesis (*GFAPD, MKI67, ASCL1, DLX6-AS1, DCX*) are changed in relation to the inflammatory status. We hypothesized that inflammation is increased in a subgroup of cases with schizophrenia and bipolar disorder and that a heightened inflammatory state will be associated with increased immune cell markers and reduced neural stem and neuronal progenitor cell markers.

## Materials and methods

### Tissue dissection, RNA extraction, cDNA synthesis and gene expression measurements

This post-mortem cohort consists of brains from 93 individuals provided by the Stanley Medical Research Institute (Rockville, USA), comprising 32 schizophrenia cases, 29 bipolar disorder cases and 32 unaffected controls. Detailed demographics are reported in Weissleder et al. [50]. The study was carried out in accordance with the Declaration of Helsinki after review at the University of New South Wales (HREC 12435, HC 17826). Methods for tissue processing, RNA extraction, complimentary DNA (cDNA) synthesis and gene expression measurements were previously described [50]. Briefly, SEZ tissue was dissected from 12 × 60 µm thick caudate sections, cut ~1.5 mm deep to the surface of the lateral ventricle. Total RNA was isolated using TRIzol as per the manufacturer’s protocol (Thermo Fisher Scientific, Carlsbad, CA, USA). cDNA was synthesised from 2 µg total RNA using the SuperScript® First-Strand Synthesis kit IV and random hexamers (Thermo Fisher Scientific). Gene expression was measured with quantitative polymerase chain reactions (qPCR) on the BioMark^TM^ HD system (Fluidigm, South San Francisco, CA, USA) or Abi Prism 7900HT fast real-time PCR (Applied Biosystems, Foster City, CA, USA) using TaqMan probes (Supplementary Table S1). Gene expression was quantified using a seven-point standard curve, and was normalised to the geometric mean of the three housekeeper genes glyceraldehyde 3-phosphate dehydrogenase (*GAPDH)*, ubiquitin C (*UBC)* and TATA-box binding protein (*TBP)*. The expression of housekeeper genes and their geometric mean did not differ by diagnosis. No template controls were run for each target gene and did not show any amplification.

### Immunohistochemistry, image acquisition and cell counting

Immunohistochemistry and counting of CD163^+^ macrophages was performed on two fresh frozen 14 μm thick sections per individual in 32 controls, 31 schizophrenia and 30 bipolar disorder cases, of which 96% of cases overlapped with the qPCR cohort [50]. In this study, CD163^+^ cell density was analysed for group differences based on the inflammatory subgroups.

### Statistical analyses

Statistical analyses were performed using IBM SPSS Statistics Version 24 (IBM, Armonk, NY, USA) and data were graphed in GraphPad Prism Version 7.02 (GraphPad, La Jolla, CA, USA). Extreme outliers were removed if they were more than two standard deviations (SD) from the mean of the diagnostic groups (2–6 individuals per target gene). Data were tested for normality using the Shapiro-Wilk test within each group. Data not fitting the Gaussian distribution were log transformed (Supplementary Information). The effects of sex and hemisphere on target gene expression were assessed (Supplementary Information). Covariates were determined with Pearson’s product-moment or Spearman’s rank correlations between each gene and age, post-mortem interval (PMI), RNA integrity number (RIN) and brain pH (Supplementary Table S2). Brain pH was not considered as a covariate in statistical analyses of group differences because brain pH is reduced in schizophrenia and bipolar disorder and associated with inflammation [73, 74]. Between-group differences were assessed using ANOVA, Welch’s ANOVA or ANCOVA; with Fisher’s least significant difference (LSD) *post hoc* tests. Semi-partial correlations were performed to control for covariates in analysis of *ICAM1* and immune cell marker gene expression as well as analysis of microglia and neurogenesis marker gene expression. Semi-partial correlation coefficients (sr) were reported. The relationships of lifetime antipsychotic dose (fluphenazine equivalent in mg), age of onset and disease duration with inflammation gene expression were analysed using Spearman’s rank correlations (Supplementary Table S3). *t*-tests or Mann–Whitney *U* tests were used to determine if gene expression differed according to history of antidepressant use (Supplementary Table S4). Fisher’s exact tests were performed to determine differences in clinical characteristics across inflammatory subgroups [i.e., sub-diagnoses, presence of psychosis, cause of death being suicide (yes, no) and evidence of peripheral inflammation (yes, no), Supplementary Information]. Since psychotic features are confounded with control and schizophrenia case status, where no controls and all schizophrenia cases had psychotic features, this was not analysed by inflammatory subgroups. Results were considered statistically significant with an α level of *p* ≤ 0.05.

A pilot two-step cluster analysis was performed using gene expression data of *IL6, IL1B, CXCL8, IL6R, IL1R1, IL6ST* and *SERPINA3*. This step was repeated removing transcripts that contributed least to the cluster until the cluster quality was +0.60, which is considered a ‘good’ silhouette measure of cohesion and separation [75]. The genes used in the final cluster solution to define high and low inflammation groups across the whole cohort were *IL1R1, SERPINA3, IL6* and *IL6R* with the respective predictor importance of 1.0, 0.46, 0.35 and 0.34 (on a scale of 0–1.0, with 1.0 being the highest importance). Five subjects with extreme outliers in two or more of the four transcripts were removed from the cluster analysis (two controls, one bipolar disorder and two schizophrenia cases). Three additional subjects had single transcript outliers removed, and those outlier values were replaced with SPSS-derived estimated means to avoid excluding additional subjects prior cluster analysis. Association between inflammation cluster group and diagnosis was assessed using Pearson’s Chi-Square test with a *post hoc* *Z*-test. The high inflammation control group only had three subjects and was therefore excluded from statistical analyses, but were displayed in graphs. Demographic details for the cohort based on inflammatory subgroups were presented in Supplementary Table S5. The effect of inflammation on immune cell count and expression of glial, immune cell and neurogenesis markers was examined for four pre-defined comparisons: (1) low inflammation schizophrenia and high inflammation schizophrenia, (2) low inflammation bipolar disorder and high inflammation bipolar disorder, (3) low inflammation control and high inflammation schizophrenia, and (4) low inflammation control and high inflammation bipolar disorder. Data were presented as gene expression relative to the mean of the control group or low inflammation control group ± standard error of the mean.

## Results

Cohort characteristics of inflammatory subgroups were described in detail in the Supplementary Information, including statistical analyses for differences in demographic variables between groups and the relationships between gene expression and demographic and clinical variables. Cohort demographics by diagnosis (controls, schizophrenia and bipolar disorder) and relationships for macrophage and neurogenesis markers were outlined in Weissleder et al. [50].

### Inflammatory marker expression was increased in the SEZ in a subgroup of cases with schizophrenia and bipolar disorder

**Fig. 1:**
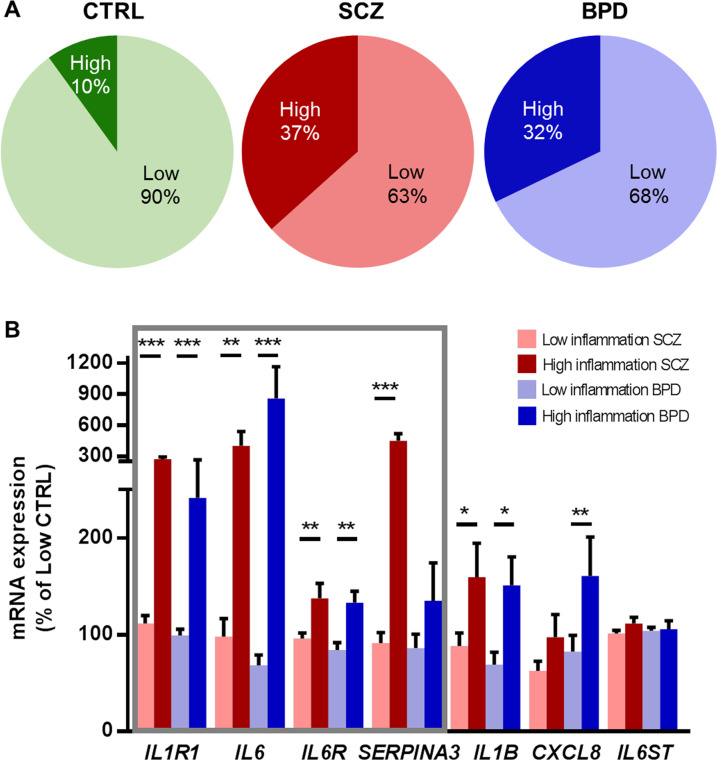
Large subgroups in schizophrenia and bipolar disorder displayed high inflammation. **A** Two-step cluster analysis of inflammatory gene expression revealed ‘high inflammation’ (total *n* = 23, darker shade) and ‘low inflammation’ (total *n* = 65, lighter shade) subgroups across the cohort. The proportion of cases in high or low inflammation subgroups significantly differed by diagnosis, with a greater proportion of schizophrenia and bipolar disorder cases in the high inflammation subgroup than controls. **B** All genes, except *IL6ST*, had significantly greater expression in high inflammation schizophrenia and bipolar disorder subgroups compared to all low inflammation subgroups. Grey box indicates the genes used in the final cluster analysis. Data are plotted relative to the mean of the low inflammation control group (100%) ± standard error of the mean. BPD bipolar disorder, CTRL control, High high inflammation, Low low inflammation, SCZ schizophrenia. *p < 0.05, **p < 0.01, ***p < 0.001.

To determine the percentage of cases defined as high and low inflammation in the SEZ in psychiatric disorders compared to unaffected controls, we measured mRNA expression of cytokines, their receptors and other inflammation-associated genes. *IL1R1* mRNA was significantly different across diagnostic groups [Supplementary Fig. S1, ANCOVA (RIN), *F*(2,84) = 4.28, *p* = 0.017], with increased expression in schizophrenia (62%, *p* = 0.004) but not in bipolar disorder compared to controls (*p* = 0.14). *IL6, IL6R, SERPINA3, IL1B, CXCL8* and *IL6ST* mRNAs did not significantly differ by diagnosis (all *p* > 0.05) although they exhibited large heterogeneity within diagnostic groups (Supplementary Fig. S1). We performed two-step cluster analysis of inflammatory gene expression. The analysis revealed two distinct groups termed ‘high inflammation’ (*n* = 23) and ‘low inflammation’ (*n* = 65) across the whole cohort. 37% of schizophrenia and 32% of bipolar disorder cases were in the high inflammation group, which were significantly greater proportions than the 10% of control cases [Pearson’s Chi-Square test, *χ*^2^ (2) = 6.29, *post hoc* *Z*-tests *p* < 0.05, Fig. 1A]. Of genes that informed the cluster, *IL1R1*, *IL6*, *IL6R* and *SERPINA3* expression was greater in the high inflammation schizophrenia and bipolar disorder subgroups compared to low inflammation subgroups (33–757%, all *p* < 0.05, Fig. 1B), except that *SERPINA3* mRNA was not elevated in high inflammation bipolar disorder compared to low inflammation controls (*p* > 0.30). In addition, *IL1B* mRNA was increased in high inflammation schizophrenia compared to low inflammation groups (59–71%, *p* < 0.035) and increased in high compared to low inflammation bipolar disorder (81%, *p* = 0.02). *CXCL8* mRNA was increased in high inflammation bipolar disorder compared to low inflammation subgroups (63–78%, *p* < 0.025).

We next assessed whether inflammatory signatures were consistent throughout the brain within the same individuals by comparing this cluster analysis result with previous findings from the dorsolateral prefrontal cortex (DLPFC) [13]. Eighty-five percent of subjects were in the same inflammatory subgroup (high or low) from both brain regions. Furthermore, the mRNA expression of *IL6*, *CXCL8*, *IL1B*, *IL1R1* and *SERPINA3* were correlated between the SEZ and the DLPFC (all rho > 0.35, all *p* < 0.001, data not shown).

### Suppression of phagocytic microglia marker gene expression in high inflammation subgroups in psychiatric disorders

**Fig. 2:**
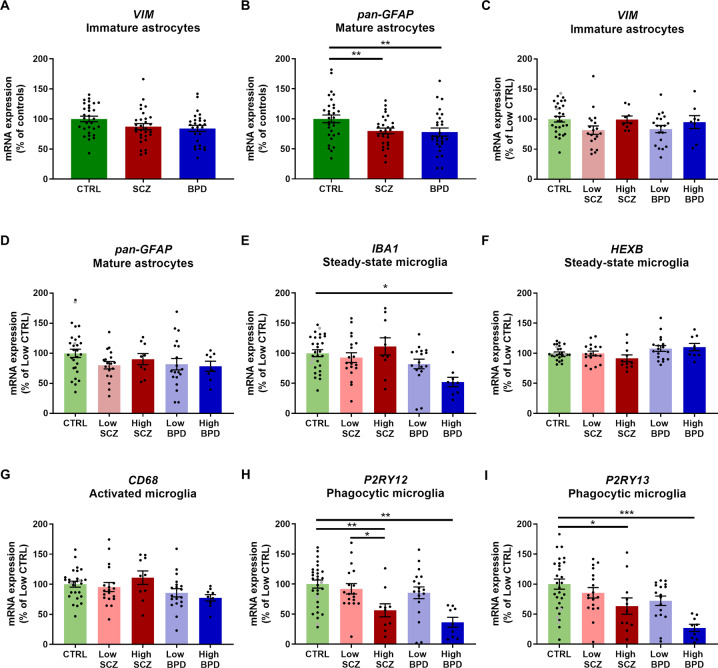
Glial cell marker gene expression was altered in diagnostic and inflammatory subgroups in psychiatric disorders. **A**–**B** *VIM* gene expression was unchanged across diagnostic groups, while *pan-GFAP* gene expression was reduced in schizophrenia and bipolar disorder compared to controls. **C**–**D** V*IM* and *pan-GFAP* mRNAs were unchanged across inflammatory subgroups. **E** *IBA1* mRNA was reduced in high inflammation bipolar disorder compared to low inflammation controls. **F**–**G** *HEXB* and *CD68* mRNAs were unchanged across inflammatory subgroups. **H**–**I** *P2RY12* and *P2RY13* mRNAs were reduced in high inflammation schizophrenia and bipolar disorder subgroups compared to low inflammation controls. Data are plotted relative to the mean of the control group (**A**–**B**) or low inflammation control group (100%) ± standard error of the mean (**C**–**I**). High inflammation controls are displayed as grey points but were not used in statistical analyses. BPD bipolar disorder, CTRL control, High high inflammation, Low low inflammation, SCZ schizophrenia. *p < 0.05, **p < 0.01, ***p < 0.001.

We next examined whether mRNAs of markers for astrocytes (*VIM*, *pan-GFAP*) and microglia (*IBA1*, *HEXB*, *CD68*, *P2RY12*, *P2RY13*) differed across diagnoses and inflammatory subgroups. Gene expression of the immature astrocyte marker *VIM* did not differ by diagnosis (*p* = 0.15, Fig. 2A). Gene expression of the mature astrocyte marker *pan-GFAP* was significantly reduced in schizophrenia and bipolar disorder compared to controls [20–22%, ANCOVA (age), *F*(2,83) = 4.99, *p* = 0.009, Fig. 2B]. Expression of the microglia markers *IBA1*, *HEXB*, *CD68* and *P2RY13* showed significant differences across diagnostic groups (Welch’s ANOVA/ ANCOVA, all *F* ≥ 4.08, all *p* ≤ 0.022, Supplementary Fig. S2). *IBA1* and *CD68* mRNAs were reduced in bipolar disorder compared to both controls (17–26%, all *p* ≤ 0.046) and schizophrenia (21–32%, all *p* ≤ 0.030). *HEXB* mRNA was increased in bipolar disorder compared to schizophrenia (14%, *p* = 0.025) and *P2RY13* mRNA was reduced in bipolar disorder compared to controls (37%, *p* = 0.003).

When analysing effects across inflammatory subgroups, *VIM*, *pan*-*GFAP*, *HEXB* and *CD68* mRNAs did not show significant differences (all *p* ≥ 0.059, Fig. 2C–D, F–G). *IBA1*, *P2RY12* and *P2RY13* mRNAs differed across inflammatory subgroups [ANCOVA, all *F* ≥ 3.60, all *p* ≤ 0.01, Fig. 2E, H–I]. *IBA1*, *P2RY12* and *P2RY13* mRNAs were all reduced in high inflammation bipolar disorder compared to low inflammation controls (48–73%, all *p* ≤ 0.015). *P2RY12* and *P2RY13* mRNAs were reduced in high inflammation schizophrenia subgroup compared to low inflammation controls (37–44%, all *p* ≤ 0.039). *P2RY12* mRNA was also reduced in high inflammation schizophrenia compared to low inflammation schizophrenia (39%, *p* = 0.019).

### Peripheral immune cell marker expression was increased in the high inflammation subgroups in psychiatric disorders

Based on the hypothesis that changes in different immune cell populations could lead to the heightened inflammatory status in psychiatric conditions, we analysed the mRNA expression of markers for macrophages (*CD163* and *CD64)*, monocytes (*CD14)*, natural killer cells (*FCGR3A)*, and an adhesion molecule important for peripheral immune cell recruitment (*ICAM1)*. We previously reported increases in *CD163* and *CD64* mRNAs in schizophrenia compared to controls and bipolar disorder [50]; however, expression of no other peripheral immune cell markers or *ICAM1* differed across diagnosis (Supplementary Fig. S3).

**Fig. 3:**
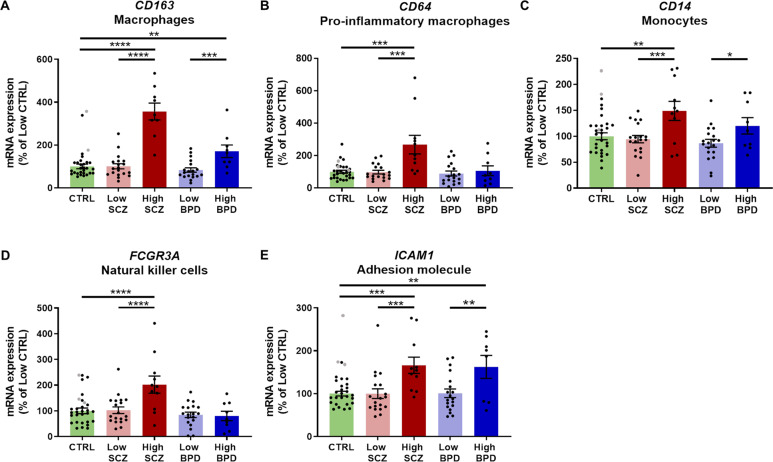
Peripheral immune cell marker and immune cell recruitment mRNA expression was increased in high inflammation subgroups in psychiatric disorders. **A**–**D** Peripheral immune cell markers had increased expression in the high inflammation schizophrenia group compared to low inflammation schizophrenia and controls. *CD163* (**A**) and *CD14* mRNAs (**C**) were also increased in high compared to low inflammation bipolar disorder. **E** *ICAM1* mRNA was increased in high compared to low inflammation groups within diagnostic groups and compared to low inflammation controls. Data are plotted relative to the mean of the low inflammation control group (100%) ± standard error of the mean. High inflammation controls are displayed as grey points but were not used in statistical analyses. BPD bipolar disorder, CTRL control, High high inflammation, Low low inflammation, SCZ schizophrenia. *p < 0.05, **p < 0.01, ***p < 0.001, ****p < 0.0001.

When analysing effects across inflammatory subgroups, all four peripheral immune cell markers *CD163, CD64, CD14, FCGR3A* and the adhesion molecule *ICAM1*, were significantly increased in high inflammation schizophrenia compared to low inflammation schizophrenia (59–254%, Welch’s ANOVA/ANOVA/ANCOVA, all *F* > 4.4, all *p* ≤ 0.003, all *post hoc* *p* ≤ 0.004, Fig. 3A–E) and low inflammation controls (49–256%, all *post hoc* *p* ≤ 0.002). We also found increased *CD163*, *CD14* and *ICAM1* mRNAs in high compared to low inflammation bipolar disorder (39–104%, all *post hoc* *p* ≤ 0.008).

### High inflammation subgroup in schizophrenia drives increased CD163^+^ macrophage density and ICAM1 may promote infiltration of immune cells

**Fig. 4:**
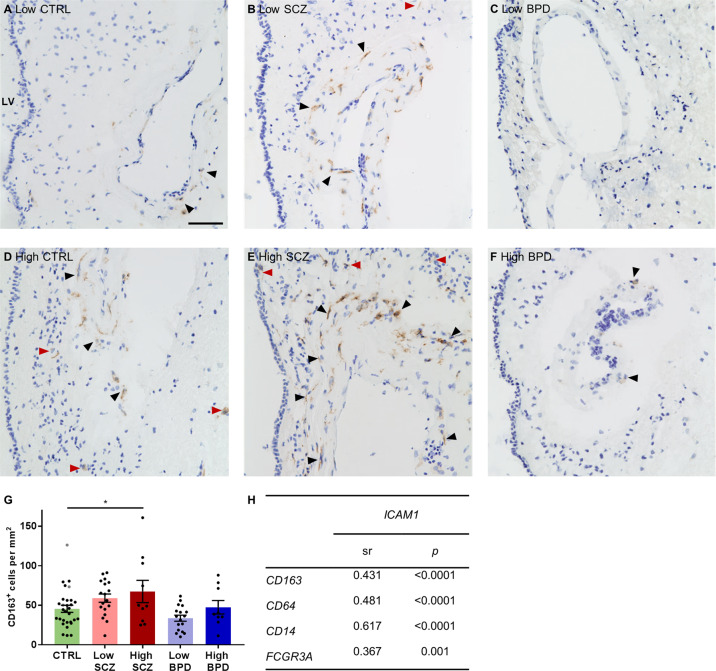
CD163^+^ macrophage density was increased in high inflammation schizophrenia and *ICAM1* correlated with peripheral immune cell marker expression. **A**–**F** Examples of CD163 immunostaining (brown) from each inflammatory subgroup with Nissl counterstaining (blue). CD163^+^ macrophages surrounded blood vessels (black arrows) and were also found in the SEZ parenchyma (red arrows). The lateral ventricle and ependymal cell layer are on the left of each image. **G** CD163^+^ macrophage numbers per mm^2^ were significantly increased in high inflammation schizophrenia compared to low inflammation controls. **H** *ICAM1* mRNA expression positively correlated with all immune cell markers. Data are plotted relative to the mean of the low inflammation control group (100%) ± standard error of the mean. High inflammation controls are displayed as grey points but were not used in statistical analyses. BPD bipolar disorder, CTRL control, High high inflammation, Low low inflammation, LV lateral ventricle, SCZ schizophrenia, sr semi-partial correlation coefficient. *p < 0.05. Scale bar = 50 μm.

We observed perivascular macrophages in the human SEZ in all subjects examined, principally along blood vessels (Fig. 4A–F). Qualitative assessment indicated that subjects with more macrophages surrounding blood vessels (Fig. 4B, D–E, black arrows) appear to have greater macrophage numbers within the parenchyma (Fig. 4B, D–E, red arrows), especially in both schizophrenia subgroups and the high inflammation control subgroup. When CD163^+^ cell density was analysed quantitatively by inflammatory subgroups, macrophage density was increased in high inflammation schizophrenia compared to low inflammation controls [Fig. 4G, 64%, ANCOVA (PMI), *F*(4,78) = 3.71, *p* = 0.008, *post hoc p* = 0.03] but did not differ significantly from low inflammation schizophrenia (*p* = 0.99), which showed an intermediate macrophage density compared to controls. CD163^+^ cell counts did not significantly differ between high inflammation bipolar disorder and low inflammation controls nor low inflammation bipolar disorder cases (all *p* > 0.73). CD163^+^ macrophages were located in the parenchyma in addition to the perivascular space indicating tissue infiltration. Therefore, we investigated the relationships between the expression of peripheral immune cell markers and *ICAM1*, one of the cell adhesion molecules that aids their transmigration from the periphery (Fig. 4H). *ICAM1* expression significantly positively correlated with *CD163*, *CD64*, *CD14* and *FCGR3A* mRNAs across diagnostic groups (all sr > 0.37, all *p* ≤ 0.001).

### Neurogenesis marker expression primarily differed by inflammatory state in schizophrenia

**Fig. 5:**
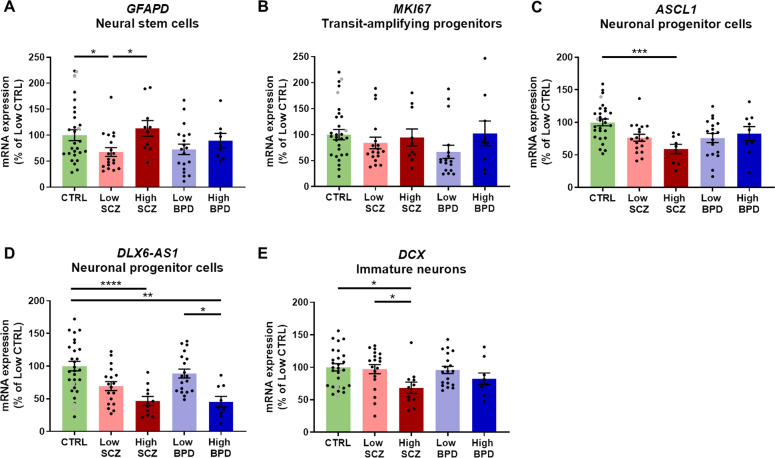
Neurogenesis marker gene expression was predominantly altered in the high inflammation subgroup in schizophrenia. **A** *GFAPD* mRNA was reduced in low inflammation schizophrenia compared to both high inflammation schizophrenia and low inflammation controls. **B** *MKI67* mRNA did not differ by inflammatory subgroup. **C** *ASCL1* mRNA was reduced in high inflammation schizophrenia compared to low inflammation controls. **D** *DLX6-AS1* mRNA was decreased in high inflammation schizophrenia compared to low inflammation controls as well as in high inflammation bipolar disorder compared to both low inflammation bipolar disorder and controls. **E** *DCX* mRNA was decreased in high inflammation schizophrenia compared to both low inflammation schizophrenia and low inflammation controls. Data are plotted relative to the mean of the low inflammation control group (100%) ± standard error of the mean. High inflammation controls are displayed as grey points but were not used in statistical analyses. BPD bipolar disorder, CTRL control, High high inflammation, Low low inflammation, SCZ schizophrenia. *p < 0.05, **p < 0.01, ***p < 0.001, ****p < 0.0001.

To determine whether the inflammatory state relates to neurogenesis marker expression in the SEZ, we compared mRNA expression of markers for quiescent neural stem cells (*GFAPD*), transit-amplifying progenitors (*MKI67*), neuronal progenitor cells (*ASCL1, DLX6-AS1*) and immature neurons (*DCX*) between inflammatory subgroups. *GFAPD* expression varied with inflammatory state, exhibiting reduced expression in low compared to high inflammation schizophrenia [67%, ANOVA, *F*(4,77) = 3.50, *p* = 0.01, *post hoc p* = 0.01, Fig. 5A]. *MKI67* mRNA did not significantly differ by inflammatory subgroup [ANOVA, *F*(4,77) = 0.90, *p* = 0.47, Fig. 5B]. *ASCL1* expression was significantly decreased in high inflammation schizophrenia compared to low inflammation controls [41%, ANOVA, *F*(4,76) = 4.98, *p* = 0.001, *post hoc* *p* < 0.0001, Fig. 5C]. *DLX6-AS1* expression was significantly decreased in high inflammation schizophrenia compared to low inflammation controls as well as high inflammation bipolar disorder compared to low inflammation bipolar disorder and low inflammation controls [49–55%, ANCOVA (RIN), *F*(4,78) = 6.44, *p* < 0.0002, all *post hoc* *p* ≤ 0.011, Fig. 5D]. Changes in *DCX* expression across inflammatory subgroups reached a trend level of statistical significance, with planned contrasts showing reduced expression in high compared to low inflammation schizophrenia [30%, ANCOVA (RIN), *F*(4,78) = 2.01, *p* = 0.10, *post hoc* *p* = 0.02] and low inflammation controls (32%, *post hoc p* = 0.01, Fig. 5E).

Since phagocytic microglia can regulate neurogenesis [76–78], we tested whether the expression of *P2RY12* and *P2RY13* were correlated with neurogenesis markers across diagnostic groups. *P2RY12* and *P2RY13* mRNAs both positively correlated with markers for neuronal progenitor cells (*ASCL1* and *DLX6*-*AS1*, all sr ≥ 0.23, all *p* ≤ 0.032) but *P2RY12* and *P2RY13* mRNAs were not correlated with any other markers for neurogenesis (*GFAPD*, *MKI67*, *DCX*, all *p* ≥ 0.16).

### Relationships between clinical variables and target gene expression in schizophrenia and bipolar disorder

The effects of psychiatric medication, age of onset and duration of illness on gene expression were assessed (Supplementary Tables S3 and S4). In schizophrenia, *IL1B* and *SERPINA3* mRNAs positively correlated and *P2RY12* mRNA negatively correlated with standardised lifetime antipsychotic dose (all *p* ≤ 0.022). Based on a dichotomous variable indicating history of antidepressant use, schizophrenia cases that had been prescribed antidepressants had lower expression of *FCGR3A* and *HEXB* mRNAs. In bipolar disorder cases that had been prescribed antidepressants, there was reduced expression of *FCGR3A*, *IBA1* and *P2RY12* mRNAs and higher expression of *HEXB* mRNA. Age of onset negatively correlated with *CD14*, *IL6R*, *IL1R1, SERPINA3, pan-GFAP* and *VIM* mRNAs in schizophrenia and *CD14*, *IL1B, SERPINA3, pan-GFAP* and *VIM* mRNAs in bipolar disorder. Duration of illness positively correlated with *IL6*, *IL6R* and *SERPINA3* mRNAs and negatively correlated with *P2RY12* mRNA in schizophrenia. Duration of illness also negatively correlated with *CXCL8* mRNA in bipolar disorder. No other significant relationships between target genes and clinical variables were detected in either schizophrenia or bipolar disorder.

## Discussion

This is the first study to define substantial subgroups of people with heightened inflammation in the largest neurogenic niche in the human brain, the SEZ. The increased percentage of those with psychiatric illness in a heightened inflammatory state is not unique to the neurogenic region but also exists in the DLPFC [13, 23], midbrain [32] and orbital frontal cortex [31]. Markers for blood-derived but not brain-resident immune cells were increased in high inflammation subgroups, suggesting peripheral immune cell infiltration represents a potential source of inflammation in schizophrenia and bipolar disorder. Supporting this, the high inflammation schizophrenia subgroup seems to drive the diagnostic change of increased macrophage density in schizophrenia compared to controls. Further, more markers relevant to various cellular developmental stages of neurogenesis differed by inflammatory subgroup in schizophrenia than in bipolar disorder, indicating putative diagnostic differences in the regulation of neurogenesis or response to inflammation.

### Peripheral immune cells may compensate for reduced microglia activity in subgroups of psychiatric disorders with elevated inflammation

The cluster analysis based on inflammatory genes expressed in the SEZ defined 32–37% of psychiatric disorder cases as high inflammation, consistent with our five previous studies in other brain regions [13, 23, 31, 32, 79]. The uniformity of inflammatory biotypes across different brain regions and an 85% overlap in inflammatory subgroup designation between the SEZ and DLPFC [13] suggests that a large subgroup of schizophrenia and bipolar disorder cases may have widespread brain inflammation. The significant association between cases in the high inflammation subgroup and evidence of peripheral inflammation corroborates that the peripheral immune system may closely interact with the brain [80]. This is supported by findings of increased communication between the periphery and the SEZ due to reduced blood-brain barrier integrity [64] as well as the identification of 48% of cases with schizophrenia displaying elevated peripheral inflammation [81]. The molecules that defined the inflammatory subgroups, *IL1R1, SERPINA3, IL6* and *IL6R*, either potentiate pro-inflammatory signalling cascades by activating transcription factors, such as nuclear factor kappa B and signal transducer and activator of transcription, or represent a state of local inflammation (*SERPINA3*). These transcription factors upregulate a variety of genes that can recruit immune cells and alter neurogenesis [82, 83].

Unlike in schizophrenia, not all blood-derived immune cell markers were increased in the high inflammation subgroup in bipolar disorder. This could signify a unique inflammatory state in bipolar disorder with potentially less pro-inflammatory macrophages, which is indicated by the lack of change in *CD64* mRNA expression. Further, expression of four microglia markers was reduced in bipolar disorder, three of which were further decreased in high inflammation bipolar disorder. This aligns with findings from Gandal et al. [84]. indicating reduced microglia marker expression in bipolar disorder. Our findings of unchanged microglia marker expression in schizophrenia compared to controls but reduced microglia marker expression in the high inflammation schizophrenia subgroup suggests that findings of reduced microglia markers across other brain regions in schizophrenia may be exacerbated if cases were classified into inflammatory subgroups [21]. Microglia have a unique phenotype in the SEZ. They are smaller and have reduced P2RY12 leading to a lack of responsiveness to ATP, which reduces phagocytosis and facilitates survival of newly generated neurons [77]. Genes essential for phagocytosis, *P2RY12* and *P2RY13*, were reduced in high inflammation subgroups, suggesting inflammation may dampen the phagocytic phenotype in the SEZ. Despite increased cytokines and increased peripheral immune cell marker expression, there was no indication of changes in microglia (*IBA1*, *HEXB*) or microglia activation markers (*CD68*). We speculate that the putative infiltration of peripheral immune cells may relate to both the lack of microglia activation and suppression of mRNA levels of markers for microglia-related phagocytosis. This hypothesis is supported by evidence from rodents where infiltration of peripheral immune cells can compensate for an abnormal microglia phenotype due to experimental knockout of *CSF1R* in microglia [85]. It is also supported by the positive correlations between blood-derived immune cell markers and the adhesion molecule *ICAM1* in this study, and gene overexpression in the diapedesis pathway in the SEZ in schizophrenia [50]. While studying post-mortem brains with psychiatric disorders is invaluable, we are unable to manipulate or monitor dynamic temporal processes such as immune cell transmigration to determine cause and effect. Therefore, while our and other research supports the potential transmigration of various immune cell types into the SEZ during inflammation [86, 87], further studies are needed to confirm this hypothesis, especially when more specific markers can be identified by single-cell studies to differentiate between brain-resident and blood-derived immune cells [88, 89].

### Inflammation primarily relates to transcripts associated with various stages of neurogenesis in schizophrenia

Investigating the inflammatory state of the SEZ has provided further insight into reduced quiescent neural stem cell (*GFAPD*) and neuronal progenitor cell marker expression (*ASCL1, DLX6-AS1*) in psychiatric disorders [50], particularly in schizophrenia. Neurogenesis marker expression changes were not as pronounced with inflammation in bipolar disorder, which may be explained by a smaller percentage of bipolar disorder cases with high inflammation, less mRNA expression of microglia and immune cell markers, and reduced density of CD163^+^ macrophages compared to schizophrenia [50]. The overall decrease in quiescent stem cell marker mRNA (*GFAPD*) in schizophrenia seems to be driven by the low inflammation subgroup. In contrast, *GFAPD* expression in the high inflammation subgroup is equivalent to controls, which may serve to maintain stem cell quiescence when inflammation is present. In the SEZ, around 20% of activated neural stem cells self-replicate producing quiescent neural stem cells, while the other 80% undergo consuming division to produce neuronal progenitor cells [90]. Our findings suggest that elevated inflammation may influence the ratio of self-replication and consuming division to favour self-replication in schizophrenia, which was initially proposed by Kalamakis et al. [91]. investigating inflammation during aging. They also demonstrate a causal link between inflammation and maintenance of neural stem cell quiescence in the rodent SEZ [91], which aligns with studies from the aging human SEZ where increased inflammation coincides with reduced proliferation and immature neuron marker expression [48, 92]. We identified that prolonged stem cell quiescence in high inflammation schizophrenia may diminish the capacity to produce neuronal progenitor cells for brain repair, which is supported by the reduction in neuronal progenitor (*ASCL1, DLX6-AS1)* and immature neuron marker expression (*DCX*). This interpretation is corroborated by increased neural stem cells quiescence along with reduced neuronal progenitor and immature neuron numbers in the rodent SEZ in response to inflammation [93, 94]. Knockout of microglial *P2RY12* leading to reduced phagocytosis decreases the number of DCX^+^/BrdU^+^ proliferating neuroblasts in rodents [76]. Therefore, significant reductions in key phagocytosis-related transcripts in the high inflammation subgroups may contribute to reductions in neuronal progenitor (*ASCL1*, *DLX6-AS1*) and immature neuron marker gene expression (*DCX*) in the SEZ. This is supported by the positive correlations between transcripts expressed by neuronal progenitor markers and *P2RY12* and *P2RY13* gene expression in this study. Animals with pre- and postnatal activation of the immune system also show decreased density of neurogenesis-associated cell types in the adult SEZ, reduced neuronal migration and decreased integration of SEZ-derived differentiated neurons in the olfactory bulb [36, 95]. The recent identification that 40% of offspring of mothers with immune system activation can be defined as having high inflammation in the midbrain [32] and periphery [96] in adulthood suggests that increasing cytokines *in utero* can recapitulate biological variation of patients with psychiatric disorders, thereby setting the stage for mechanistic studies of neurogenesis after prenatal immune activation with the possibility of stratifying based on inflammatory status. Moreover, increased IL6 reduces human hippocampal DCX^+^ immature neurons through apoptosis in vitro [97], implying that newly generated neurons may undergo apoptosis in the SEZ in the high inflammation subgroup of schizophrenia. The magnitude of change in neurogenesis because of increased inflammation and immune cells is unclear in the human SEZ; however, studies in rodents indicate a 38% increase in neural stem cells and 36% increase in cell proliferation when depleting natural killer cells [87], suggesting a direct causal relationship with neurogenesis.

Reduced *DCX* expression in high inflammation schizophrenia could also represent increased migration of immature neurons out of the SEZ, which may relate to increased interstitial white matter neuron density in schizophrenia [98, 99]. Heightened inflammation in the DLPFC is associated with an increased density of interstitial white matter neurons [100] and reduced expression of inhibitory interneuron markers (glutamate decarboxylase 67, somatostatin and parvalbumin) [23, 101]. Inhibitory interneurons from the SEZ are proposed to migrate and functionally integrate into both the prefrontal cortex [58] and striatum [38], two regions where schizophrenia neuropathology is thought to underlie symptomatology. While the relationships between impaired neurogenesis and inhibitory interneuron deficits in the prefrontal cortex and striatum remain to be examined, decreased *ASCL1*, *DLX6-AS1* and *DCX* gene expression in high inflammation subgroups pinpoints altered regulation of neurogenesis in the SEZ as a possible origin of the widely reported inhibitory interneuron deficits in psychiatric disorders [10, 102]. This is also supported by the reduced density of calretinin-positive interneurons in the caudate nucleus in schizophrenia [103].

### Relationships between clinical variables, inflammation and neurogenesis marker expression in schizophrenia and bipolar disorder

The effects of antipsychotics vary depending on their type and dose [104, 105], with in vitro studies demonstrating both anti- and pro-inflammatory effects [104]. Antipsychotics can reduce the expression of pro-inflammatory cytokines in the periphery such as IL1β, IL6 and TGFβ [28, 106]. However, regardless of prolonged antipsychotic treatment, we identified both a subgroup of cases with psychiatric disorders who displayed elevated inflammation and positive correlations between both *IL1B* and *SERPINA3* mRNAs and lifetime antipsychotic dose. While chronic administration of antipsychotics in rodents can increase microglia activation and proliferation [107], others find that treatment of rodent microglia with antipsychotics reduces the synthesis of pro-inflammatory cytokines and stimulus-induced inflammatory signalling [108]. We found no association between the marker for microglia activation, *CD68*, and lifetime antipsychotic dose. However, the negative correlation between lifetime antipsychotic dose and *P2RY12* mRNA in schizophrenia suggests that antipsychotics may relate to reduced microglia-mediated phagocytosis in the SEZ. These results support the possibility that antipsychotics may change the microglia phenotype in people with schizophrenia. There are several other observations that support the theory that elevated inflammation is not necessarily solely the consequence of antipsychotic exposure. Firstly, higher inflammation correlates with greater symptom severity in drug naïve ‘ultra-high risk’ schizophrenia patients [109]. Secondly, inflammatory pathways are unchanged in the cortex of antipsychotic-exposed monkeys [110]. Thirdly, inflammation is elevated in both medicated and un-medicated patients prior to death [111]. Finally, a meta-analysis shows decreased circulating IL6 levels after antipsychotic treatment in schizophrenia patients [12]. While we cannot be certain that increased inflammation is not a consequence of antipsychotic treatment, the current literature implies that increased antipsychotic doses may be a consequence of the detrimental effects of inflammation on symptoms.

Other medications, such as antidepressants, as well as clinical features, including mode of death, may also relate to inflammation or neurogenesis. Antidepressant medication can have anti-inflammatory effects [112], which aligns with our findings of reduced mRNA levels of the natural killer cell marker *FCGR3A* and the microglia markers *IBA1* and *P2RY12* in psychiatric disorder cases with a history of antidepressant use. Although antidepressants enhance neurogenesis in the hippocampus [113, 114], their effects are less clear in the SEZ [115]. In this cohort, a history of antidepressant use was not associated with changes in neurogenesis marker expression [50]. Despite the potentially anti-inflammatory and pro-neurogenic effects of antidepressants on some targets of interest, we still found elevated inflammation and its relationship with altered neurogenesis. The increased percentage of bipolar disorder cases with psychosis in the high inflammatory subgroup aligns with research showing a relationship between inflammation and positive symptoms [116], and elevated inflammation at first episode of psychosis [117, 118]. Our finding that the suicide status was associated with the low inflammation subgroup adds to the already conflicting literature showing either no relationships [119] or positive relationships between suicide and expression of pro-inflammatory [120] and anti-inflammatory cytokines [121]. Since inflammation may relate to clinical outcomes, the role of inflammation in suicide warrants further exploration in psychiatric disorders.

## Conclusion

High inflammation subgroups in psychiatric disorders were associated with increased mRNAs of markers for peripheral immune cells and increased macrophage density in schizophrenia. Further, the correlations of immune cell markers with *ICAM1* suggest potential transmigration of immune cells from the peripheral vasculature into the SEZ. This study also discovered that the relationship between inflammation and alterations in transcripts associated with various cellular developmental stages of neurogenesis is more pronounced in schizophrenia compared to bipolar disorder. The juxtaposing relationship between inflammation and quiescent neural stem cell marker restoration as opposed to reductions in immature neuron markers demonstrates a complexity that may be attributed to the balance of immune cells and inflammatory cytokines. Our findings set the scene for further experimental research that will be crucial to understand the mechanisms by which inflammation and immune cell recruitment alters neurogenesis across the course of psychiatric disorders, which may enable us to harness the vast potential of neurogenesis.

## Supplementary information

Supplementary Information

Supplementary Tables

## References

[1] Perälä J, Suvisaari J, Saarni SI, Kuoppasalmi K, Isometsä E, Pirkola S (2007). Lifetime prevalence of psychotic and bipolar I disorders in a general population. Arch Gen Psychiatry.

[2] Merikangas KR, Jin R, He JP, Kessler RC, Lee S, Sampson NA (2011). Prevalence and correlates of bipolar spectrum disorder in the world mental health survey initiative. Arch Gen Psychiatry.

[3] Keck PE, McElroy SL, Havens JR, Altshuler LL, Nolen WA, Frye MA (2003). Psychosis in bipolar disorder: Phenomenology and impact on morbidity and course of illness. Compr Psychiatry.

[4] Vöhringer PA, Barroilhet SA, Amerio A, Reale ML, Alvear K, Vergne D (2013). Cognitive impairment in bipolar disorder and schizophrenia: a systematic review. Front Psychiatry.

[5] Daneluzzo E, Arduini L, Rinaldi O, Di Domenico M, Petruzzi C, Kalyvoka A (2002). PANSS factors and scores in schizophrenic and bipolar disorders during an index acute episode: a further analysis of the cognitive component. Schizophrenia Res.

[6] Lichtenstein P, Yip BH, Björk C, Pawitan Y, Cannon TD, Sullivan PF (2009). Common genetic determinants of schizophrenia and bipolar disorder in Swedish families: a population-based study. Lancet.

[7] Bramon E, Sham PC (2001). The common genetic liability between schizophrenia and bipolar disorder: a review. Curr Psychiatry Rep..

[8] Cross-Disorder Group of the Psychiatric Genomics Consortium. (2013). Genetic relationship between five psychiatric disorders estimated from genome-wide SNPs. Nat Genet.

[9] Benes FM, Berretta S (2001). GABAergic interneurons: implications for understanding schizophrenia and bipolar disorder. Neuropsychopharmacology.

[10] Fung SJ, Fillman SG, Webster MJ, Shannon Weickert C (2014). Schizophrenia and bipolar disorder show both common and distinct changes in cortical interneuron markers. Schizophr Res.

[11] Lewis DA, Curley AA, Glausier JR, Volk DW (2012). Cortical parvalbumin interneurons and cognitive dysfunction in schizophrenia. Trends Neurosci.

[12] Goldsmith DR, Rapaport MH, Miller BJ (2016). A meta-analysis of blood cytokine network alterations in psychiatric patients: comparisons between schizophrenia, bipolar disorder and depression. Mol Psychiatry.

[13] Fillman SG, Sinclair D, Fung SJ, Webster MJ, Shannon Weickert C (2014). Markers of inflammation and stress distinguish subsets of individuals with schizophrenia and bipolar disorder. Transl Psychiatry.

[14] Lanz TA, Reinhart V, Sheehan MJ, Rizzo S, Bove SE, James LC (2019). Postmortem transcriptional profiling reveals widespread increase in inflammation in schizophrenia: a comparison of prefrontal cortex, striatum, and hippocampus among matched tetrads of controls with subjects diagnosed with schizophrenia, bipolar or major depressive disorder. Transl Psychiatry.

[15] North HF, Bruggemann J, Cropley V, Swaminathan V, Sundram S, Lenroot R (2021). Increased peripheral inflammation in schizophrenia is associated with worse cognitive performance and related cortical thickness reductions. Eur Arch Psychiatry Clin Neurosci.

[16] Leboyer M, Soreca I, Scott J, Frye M, Henry C, Tamouza R (2012). Can bipolar disorder be viewed as a multi-system inflammatory disease?. J Affect Disord.

[17] Knuesel I, Chicha L, Britschgi M, Schobel SA, Bodmer M, Hellings JA (2014). Maternal immune activation and abnormal brain development across CNS disorders. Nat Rev Neurol.

[18] Schizophrenia Working Group of the Psychiatric Genomics Consortium. (2014). Biological insights from 108 schizophrenia-associated genetic loci. Nature.

[19] van Kesteren CF, Gremmels H, de Witte LD, Hol EM, Van Gool AR, Falkai PG (2017). Immune involvement in the pathogenesis of schizophrenia: a meta-analysis on postmortem brain studies. Transl Psychiatry.

[20] Trepanier MO, Hopperton KE, Mizrahi R, Mechawar N, Bazinet RP (2016). Postmortem evidence of cerebral inflammation in schizophrenia: a systematic review. Mol Psychiatry.

[21] Snijders G, van Zuiden W, Sneeboer M, Berdenis van Berlekom A, van der Geest AT, Schnieder T (2021). A loss of mature microglial markers without immune activation in schizophrenia. Glia.

[22] Sneeboer MAM, Snijders G, Berdowski WM, Fernández-Andreu A, van Mierlo HC, Psychiatric Donor Program of the Netherlands Brain Bank (2019). Microglia in post-mortem brain tissue of patients with bipolar disorder are not immune activated. Transl Psychiatry.

[23] Fillman SG, Cloonan N, Catts VS, Miller LC, Wong J, McCrossin T (2013). Increased inflammatory markers identified in the dorsolateral prefrontal cortex of individuals with schizophrenia. Mol Psychiatry.

[24] Volk DW, Chitrapu A, Edelson JR, Roman KM, Moroco AE, Lewis DA (2015). Molecular mechanisms and timing of cortical immune activation in schizophrenia. Am J Psychiatry.

[25] Rao JS, Harry GJ, Rapoport SI, Kim HW (2010). Increased excitotoxicity and neuroinflammatory markers in postmortem frontal cortex from bipolar disorder patients. Mol Psychiatry.

[26] Nassar A, Azab AN (2014). Effects of lithium on inflammation. Acs Chem Neurosci.

[27] Juncal-Ruiz M, Riesco-Dávila L, Ortiz-García de la Foz V, Martínez-Garcia O, Ramírez-Bonilla M, Ocejo-Viñals JG (2018). Comparison of the anti-inflammatory effect of aripiprazole and risperidone in 75 drug-naive first episode psychosis individuals: a 3 months randomized study. Schizophr Res.

[28] Miller BJ, Buckley P, Seabolt W, Mellor A, Kirkpatrick B (2011). Meta-analysis of cytokine alterations in schizophrenia: clinical status and antipsychotic effects. Biol Psychiatry.

[29] Volk DW, Sampson AR, Zhang Y, Edelson JR, Lewis DA (2016). Cortical GABA markers identify a molecular subtype of psychotic and bipolar disorders. Psychol Med.

[30] Schwarz E, van Beveren NJ, Ramsey J, Leweke FM, Rothermundt M, Bogerts B (2014). Identification of subgroups of schizophrenia patients with changes in either immune or growth factor and hormonal pathways. Schizophr Bull.

[31] Zhang Y, Catts VS, Sheedy D, McCrossin T, Kril JJ, Shannon Weickert C (2016). Cortical grey matter volume reduction in people with schizophrenia is associated with neuro-inflammation. Transl Psychiatry.

[32] Purves-Tyson TD, Weber-Stadlbauer U, Richetto J, Rothmond DA, Labouesse MA, Polesel M (2019). Increased levels of midbrain immune-related transcripts in schizophrenia and in murine offspring after maternal immune activation. Mol Psychiatry.

[33] Cai HQ, Catts VS, Webster MJ, Galletly C, Liu D, O’Donnell M (2020). Increased macrophages and changed brain endothelial cell gene expression in the frontal cortex of people with schizophrenia displaying inflammation. Mol Psychiatry.

[34] Catts VS, Wong J, Fillman SG, Fung SJ, Shannon Weickert C (2014). Increased expression of astrocyte markers in schizophrenia: association with neuroinflammation. Aust N. Z J Psychiatry.

[35] Ekdahl CT, Kokaia Z, Lindvall O (2009). Brain inflammation and adult neurogenesis: the dual role of microglia. Neuroscience.

[36] Liu YH, Lai WS, Tsay HJ, Wang TW, Yu JY (2013). Effects of maternal immune activation on adult neurogenesis in the subventricular zone-olfactory bulb pathway and olfactory discrimination. Schizophr Res.

[37] Curtis MA, Low VF, Faull RL (2012). Neurogenesis and progenitor cells in the adult human brain: a comparison between hippocampal and subventricular progenitor proliferation. Dev Neurobiol.

[38] Ernst A, Alkass K, Bernard S, Salehpour M, Perl S, Tisdale J (2014). Neurogenesis in the striatum of the adult human brain. Cell.

[39] Iannitelli A, Quartini A, Tirassa P, Bersani G (2017). Schizophrenia and neurogenesis: a stem cell approach. Neurosci Biobehav Rev.

[40] Weissleder C, North HF, Shannon Weickert C (2019). Important unanswered questions about adult neurogenesis in schizophrenia. Curr Opin Psychiatry.

[41] Shaw AD, Tiwari Y, Kaplan W, Heath A, Mitchell PB, Schofield PR (2014). Characterisation of genetic variation in ST8SIA2 and its interaction region in NCAM1 in patients with bipolar disorder. PLoS ONE.

[42] Le Strat Y, Ramoz N, Gorwood P (2009). The role of genes involved in neuroplasticity and neurogenesis in the observation of a gene-environment interaction (GxE) in schizophrenia. Curr Mol Med.

[43] Hayashi Y, Jinnou H, Sawamoto K, Hitoshi S (2018). Adult neurogenesis and its role in brain injury and psychiatric diseases. J Neurochem.

[44] Sorrells SF, Paredes MF, Cebrian-Silla A, Sandoval K, Qi D, Kelley KW (2018). Human hippocampal neurogenesis drops sharply in children to undetectable levels in adults. Nature.

[45] Dennis CV, Suh LS, Rodriguez ML, Kril JJ, Sutherland GT (2016). Human adult neurogenesis across the ages: an immunohistochemical study. Neuropathol Appl Neurobiol.

[46] Moreno-Jiménez EP, Flor-García M, Terreros-Roncal J, Rábano A, Cafini F, Pallas-Bazarra N (2019). Adult hippocampal neurogenesis is abundant in neurologically healthy subjects and drops sharply in patients with Alzheimer’s disease. Nat Med.

[47] Boldrini M, Fulmore CA, Tartt AN, Simeon LR, Pavlova I, Poposka V (2018). Human Hippocampal Neurogenesis Persists throughout Aging. Cell Stem Cell.

[48] Weissleder C, Fung SJ, Wong MW, Barry G, Double KL, Halliday GM (2016). Decline in proliferation and immature neuron markers in the human subependymal zone during aging: relationship to EGF- and FGF-related transcripts. Front Aging Neurosci.

[49] Weickert CS, Webster MJ, Colvin SM, Herman MM, Hyde TM, Weinberger DR (2000). Localization of epidermal growth factor receptors and putative neuroblasts in human subependymal zone. J Comp Neurol.

[50] Weissleder C, North HF, Bitar M, Fullerton JM, Sager R, Barry G, et al. Reduced adult neurogenesis is associated with increased macrophages in the subependymal zone in schizophrenia. Mol Psychiatry. 2021. 10.1038/s41380-021-01149-3. 10.1038/s41380-021-01149-3 34059796

[51] Quiñones-Hinojosa A, Sanai N, Soriano-Navarro M, Gonzalez-Perez O, Mirzadeh Z, Gil-Perotin S (2006). Cellular composition and cytoarchitecture of the adult human subventricular zone: a niche of neural stem cells. J Comp Neurol.

[52] van den Berge SA, Middeldorp J, Zhang CE, Curtis MA, Leonard BW, Mastroeni D (2010). Longterm quiescent cells in the aged human subventricular neurogenic system specifically express GFAP-delta. Aging Cell.

[53] Zhang J, Jiao J (2015). Molecular biomarkers for embryonic and adult neural stem cell and neurogenesis. Biomed Res Int.

[54] Kim EJ, Ables JL, Dickel LK, Eisch AJ, Johnson JE (2011). Ascl1 (Mash1) defines cells with long-term neurogenic potential in subgranular and subventricular zones in adult mouse brain. PLoS ONE.

[55] Dulken BW, Leeman DS, Boutet SC, Hebestreit K, Brunet A (2017). Single-cell transcriptomic analysis defines heterogeneity and transcriptional dynamics in the adult neural stem cell lineage. Cell Rep..

[56] Curtis MA, Kam M, Nannmark U, Anderson MF, Axell MZ, Wikkelso C (2007). Human neuroblasts migrate to the olfactory bulb via a lateral ventricular extension. Science.

[57] Inta D, Alfonso J, von Engelhardt J, Kreuzberg MM, Meyer AH, van Hooft JA (2008). Neurogenesis and widespread forebrain migration of distinct GABAergic neurons from the postnatal subventricular zone. Proc Natl Acad Sci USA.

[58] Paredes MF, James D, Gil-Perotin S, Kim H, Cotter JA, Ng C, et al. Extensive migration of young neurons into the infant human frontal lobe. Science. 2016;354:aaf7073. 10.1126/science.aaf7073 PMC5436574 27846470

[59] Weissleder C, Webster MJ, Barry G, Shannon Weickert C. Reduced insulin like growth factor family member expression predicts neurogenesis marker expression in the subependymal zone in schizophrenia and bipolar disorder. Schizophr Bull. 2021;47:1168–78. 10.1093/schbul/sbaa159 PMC8266571 33274367

[60] Borsini A, Zunszain PA, Thuret S, Pariante CM (2015). The role of inflammatory cytokines as key modulators of neurogenesis. Trends Neurosci.

[61] Galic MA, Riazi K, Pittman QJ (2012). Cytokines and brain excitability. Front Neuroendocrinol.

[62] Covacu R, Arvidsson L, Andersson A, Khademi M, Erlandsson-Harris H, Harris RA (2009). TLR activation induces TNF-alpha production from adult neural stem/progenitor cells. J Immunol.

[63] Banks WA, Kastin AJ, Broadwell RD (1995). Passage of cytokines across the blood-brain barrier. Neuroimmunomodulation.

[64] Tavazoie M, Van der Veken L, Silva-Vargas V, Louissaint M, Colonna L, Zaidi B (2008). A specialized vascular niche for adult neural stem cells. Cell Stem Cell.

[65] Lecuyer MA, Kebir H, Prat A (2016). Glial influences on BBB functions and molecular players in immune cell trafficking. Biochim Biophys Acta.

[66] Koo JW, Russo SJ, Ferguson D, Nestler EJ, Duman RS (2010). Nuclear factor-kappaB is a critical mediator of stress-impaired neurogenesis and depressive behavior. Proc Natl Acad Sci USA.

[67] Fan LW, Pang Y (2017). Dysregulation of neurogenesis by neuroinflammation: key differences in neurodevelopmental and neurological disorders. Neural Regen Res.

[68] Butovsky O, Ziv Y, Schwartz A, Landa G, Talpalar AE, Pluchino S (2006). Microglia activated by IL-4 or IFN-gamma differentially induce neurogenesis and oligodendrogenesis from adult stem/progenitor cells. Mol Cell Neurosci.

[69] Hu X, Leak RK, Shi Y, Suenaga J, Gao Y, Zheng P (2015). Microglial and macrophage polarization-new prospects for brain repair. Nat Rev Neurol.

[70] Ekdahl CT, Claasen JH, Bonde S, Kokaia Z, Lindvall O (2003). Inflammation is detrimental for neurogenesis in adult brain. Proc Natl Acad Sci USA.

[71] Kim WK, Alvarez X, Fisher J, Bronfin B, Westmoreland S, McLaurin J (2006). CD163 identifies perivascular macrophages in normal and viral encephalitic brains and potential precursors to perivascular macrophages in blood. Am J Pathol.

[72] Ritter M, Buechler C, Kapinsky M, Schmitz G (2001). Interaction of CD163 with the regulatory subunit of casein kinase II (CKII) and dependence of CD163 signaling on CKII and protein kinase C. Eur J Immunol.

[73] Hagihara H, Catts VS, Katayama Y, Shoji H, Takagi T, Huang FL (2018). Decreased brain pH as a shared endophenotype of psychiatric disorders. Neuropsychopharmacology.

[74] Reeh PW, Steen KH. Chapter 8. Tissue acidosis in nociception and pain. *The Polymodal Pathological Pain Receptor—A Gateway to Pathological Pain*, 1996, pp 143–51. 10.1016/s0079-6123(08)61085-7 9009732

[75] Rousseeuw PJ (1987). Silhouettes - a graphical aid to the interpretation and validation of cluster-analysis. J Computational Appl Math.

[76] Diaz-Aparicio I, Paris I, Sierra-Torre V, Plaza-Zabala A, Rodríguez-Iglesias N, Márquez-Ropero M (2020). Microglia actively remodel adult hippocampal neurogenesis through the phagocytosis secretome. J Neurosci.

[77] Ribeiro Xavier AL, Kress BT, Goldman SA, Lacerda de Menezes JR, Nedergaard M (2015). A distinct population of microglia supports adult neurogenesis in the subventricular zone. J Neurosci.

[78] Sierra A, Encinas JM, Deudero JJ, Chancey JH, Enikolopov G, Overstreet-Wadiche LS (2010). Microglia shape adult hippocampal neurogenesis through apoptosis-coupled phagocytosis. Cell Stem Cell.

[79] Fillman SG, Weickert TW, Lenroot RK, Catts SV, Bruggemann JM, Catts VS (2016). Elevated peripheral cytokines characterize a subgroup of people with schizophrenia displaying poor verbal fluency and reduced Broca’s area volume. Mol Psychiatry.

[80] Lampa J, Westman M, Kadetoff D, Agréus AN, Le Maître E, Gillis-Haegerstrand C (2012). Peripheral inflammatory disease associated with centrally activated IL-1 system in humans and mice. Proc Natl Acad Sci USA.

[81] Boerrigter D, Weickert TW, Lenroot R, O’Donnell M, Galletly C, Liu D (2017). Using blood cytokine measures to define high inflammatory biotype of schizophrenia and schizoaffective disorder. J Neuroinflammation.

[82] Zhang Y, Hu W (2012). NFkappaB signaling regulates embryonic and adult neurogenesis. Front Biol.

[83] Gu F, Hata R, Ma YJ, Tanaka J, Mitsuda N, Kumon Y (2005). Suppression of Stat3 promotes neurogenesis in cultured neural stem cells. J Neurosci Res.

[84] Gandal MJ, Zhang P, Hadjimichael E, Walker RL, Chen C, Liu S, et al. Transcriptome-wide isoform-level dysregulation in ASD, schizophrenia, and bipolar disorder. Science. 2018;362:eaat8127. 10.1126/science.aat8127 PMC6443102 30545856

[85] Pons V, Laflamme N, Prefontaine P, Rivest S (2020). Role of macrophage colony-stimulating factor receptor on the proliferation and survival of microglia following systemic nerve and cuprizone-induced injuries. Front Immunol.

[86] Ousman SS, Kubes P (2012). Immune surveillance in the central nervous system. Nat Neurosci.

[87] Liu Q, Sanai N, Jin WN, La Cava A, Van Kaer L, Shi FD (2016). Neural stem cells sustain natural killer cells that dictate recovery from brain inflammation. Nat Neurosci.

[88] Bennett FC, Bennett ML, Yaqoob F, Mulinyawe SB, Grant GA, Hayden Gephart M (2018). A combination of ontogeny and CNS environment establishes microglial identity. Neuron.

[89] Prinz M, Priller J (2014). Microglia and brain macrophages in the molecular age: from origin to neuropsychiatric disease. Nat Rev Neurosci.

[90] Obernier K, Cebrian-Silla A, Thomson M, Parraguez JI, Anderson R, Guinto C (2018). Adult neurogenesis is sustained by symmetric self-renewal and differentiation. Cell Stem Cell.

[91] Kalamakis G, Brüne D, Ravichandran S, Bolz J, Fan W, Ziebell F (2019). Quiescence modulates stem cell maintenance and regenerative capacity in the aging brain. Cell.

[92] Barry G, Guennewig B, Fung S, Kaczorowski D, Weickert CS (2015). Long non-coding RNA expression during aging in the human subependymal zone. Front Neurol.

[93] Belenguer G, Duart-Abadia P, Jordán-Pla A, Domingo-Muelas A, Blasco-Chamarro L, Ferrón SR (2021). Adult neural stem cells are alerted by systemic inflammation through TNF-alpha receptor signaling. Cell Stem Cell.

[94] Pluchino S, Muzio L, Imitola J, Deleidi M, Alfaro-Cervello C, Salani G (2008). Persistent inflammation alters the function of the endogenous brain stem cell compartment. Brain.

[95] Tepavčević V, Lazarini F, Alfaro-Cervello C, Kerninon C, Yoshikawa K, Garcia-Verdugo JM (2011). Inflammation-induced subventricular zone dysfunction leads to olfactory deficits in a targeted mouse model of multiple sclerosis. J Clin Invest.

[96] Mueller FS, Scarborough J, Schalbetter SM, Richetto J, Kim E, Couch A (2021). Behavioral, neuroanatomical, and molecular correlates of resilience and susceptibility to maternal immune activation. Mol Psychiatry.

[97] Borsini A, Cattaneo A, Malpighi C, Thuret S, Harrison NA, MRC ImmunoPsychiatry C. (2018). Interferon-alpha reduces human hippocampal neurogenesis and increases apoptosis via activation of distinct STAT1-dependent mechanisms. Int J Neuropsychopharmacol.

[98] Yang Y, Fung SJ, Rothwell A, Tianmei S, Weickert CS (2011). Increased interstitial white matter neuron density in the dorsolateral prefrontal cortex of people with schizophrenia. Biol Psychiatry.

[99] Joshi D, Fung SJ, Rothwell A, Weickert CS (2012). Higher gamma-aminobutyric acid neuron density in the white matter of orbital frontal cortex in schizophrenia. Biol Psychiatry.

[100] Fung SJ, Joshi D, Fillman SG, Weickert CS (2014). High white matter neuron density with elevated cortical cytokine expression in schizophrenia. Biol Psychiatry.

[101] Falco A, Pennucci R, Brambilla E, de Curtis I (2014). Reduction in parvalbumin-positive interneurons and inhibitory input in the cortex of mice with experimental autoimmune encephalomyelitis. Exp Brain Res.

[102] Dienel SJ, Lewis DA (2019). Alterations in cortical interneurons and cognitive function in schizophrenia. Neurobiol Dis.

[103] Adorjan I, Sun B, Feher V, Tyler T, Veres D, Chance SA (2020). Evidence for decreased density of calretinin-immunopositive neurons in the caudate nucleus in patients with schizophrenia. Front Neuroanat.

[104] Baumeister D, Ciufolini S, Mondelli V (2016). Effects of psychotropic drugs on inflammation: consequence or mediator of therapeutic effects in psychiatric treatment?. Psychopharmacology.

[105] Dinesh AA, Islam J, Khan J, Turkheimer F, Vernon AC (2020). Effects of antipsychotic drugs: cross talk between the nervous and innate immune system. CNS Drugs.

[106] Feng T, McEvoy JP, Miller BJ (2020). Longitudinal study of inflammatory markers and psychopathology in schizophrenia. Schizophr Res.

[107] Cotel MC, Lenartowicz EM, Natesan S, Modo MM, Cooper JD, Williams SC (2015). Microglial activation in the rat brain following chronic antipsychotic treatment at clinically relevant doses. Eur Neuropsychopharmacol.

[108] Monji A, Kato T, Kanba S (2009). Cytokines and schizophrenia: Microglia hypothesis of schizophrenia. Psychiatry Clin Neurosci.

[109] Bloomfield PS, Selvaraj S, Veronese M, Rizzo G, Bertoldo A, Owen DR (2016). Microglial activity in people at ultra high risk of psychosis and in schizophrenia: an [(11)C]PBR28 PET brain imaging study. Am J Psychiatry.

[110] Volk DW, Moroco AE, Roman KM, Edelson JR, Lewis DA (2019). The role of the nuclear factor-kappaB transcriptional complex in cortical immune activation in schizophrenia. Biol Psychiatry.

[111] Saetre P, Emilsson L, Axelsson E, Kreuger J, Lindholm E, Jazin E (2007). Inflammation-related genes up-regulated in schizophrenia brains. BMC Psychiatry.

[112] Kalkman HO, Feuerbach D (2016). Antidepressant therapies inhibit inflammation and microglial M1-polarization. Pharmacol Therapeutics.

[113] Anacker C, Zunszain PA, Cattaneo A, Carvalho LA, Garabedian MJ, Thuret S (2011). Antidepressants increase human hippocampal neurogenesis by activating the glucocorticoid receptor. Mol Psychiatry.

[114] Boldrini M, Hen R, Underwood MD, Rosoklija GB, Dwork AJ, Mann JJ (2012). Hippocampal angiogenesis and progenitor cell proliferation are increased with antidepressant use in major depression. Biol Psychiatry.

[115] Ohira K, Miyakawa T (2011). Chronic treatment with fluoxetine for more than 6 weeks decreases neurogenesis in the subventricular zone of adult mice. Mol Brain.

[116] Liemburg E, Nolte I, Klein H, Knegtering H (2018). Relation of inflammatory markers with symptoms of psychotic disorders: a large cohort study. Prog Neuro-Psychopharmacol Biol Psychiatry.

[117] Pardo-de-Santayana G, Juncal-Ruiz M, Vázquez-Bourgon J, Riesco-Dávila L, Ortiz-Garcia de la Foz V, Pelayo-Terán JM (2021). Active psychosis and pro-inflammatory cytokines in first-episode of psychosis. J Psychiatr Res.

[118] Fraguas D, Díaz-Caneja CM, Ayora M, Hernández-Álvarez F, Rodríguez-Quiroga A, Recio S (2019). Oxidative stress and inflammation in first-episode psychosis: a systematic review and meta-analysis. Schizophrenia Bull.

[119] Torres-Platas SG, Cruceanu C, Chen GG, Turecki G, Mechawar N (2014). Evidence for increased microglial priming and macrophage recruitment in the dorsal anterior cingulate white matter of depressed suicides. Brain, Behav, Immun.

[120] Pandey GN, Rizavi HS, Ren X, Fareed J, Hoppensteadt DA, Roberts RC (2012). Proinflammatory cytokines in the prefrontal cortex of teenage suicide victims. J Psychiatr Res.

[121] Tonelli LH, Stiller J, Rujescu D, Giegling I, Schneider B, Maurer K (2008). Elevated cytokine expression in the orbitofrontal cortex of victims of suicide. Acta Psychiatr Scandinavica.

