# Supplementary material for: A schizophrenia subgroup with elevated inflammation displays reduced microglia, increased peripheral immune cell and altered neurogenesis marker gene expression in the subependymal zone
Source: Transl Psychiatry. 2021 Dec 15;11:635. doi: 10.1038/s41398-021-01742-8 (PMC8674325; doi:10.1038/s41398-021-01742-8)
Supplement: Supplementary file 1 — Supplementary Information [file 41398_2021_1742_MOESM1_ESM.docx]

# SUPPLEMENTARY INFORMATION

*Corresponding author:

Cynthia Shannon Weickert

Neuroscience Research Australia

Schizophrenia Research Laboratory

Margarete Ainsworth Building, 139 Barker Street

Randwick, NSW, Australia, 2031

Phone: +61 2 9399 1717

Email: weickerc@upstate.edu

**Analysis of demographic variables in the post-mortem cohort**

Details for the cohort demographics by diagnosis (controls, schizophrenia and bipolar disorder) were outlined previously.^1^ **Supplementary Table S5** details the demographics and clinical variables for this cohort based on inflammatory subgroups. Inflammatory subgroups did not differ according to age at death, RIN, PMI, hemisphere or smoking at time of death (all *p*>0.05, data not shown). Brain pH was significantly lower in high inflammation schizophrenia compared to low inflammation controls (*p*=0.01), and in high inflammation bipolar disorder compared to both low inflammation controls (*p*=0.001) and low inflammation bipolar disorder (*p*=0.04). There was a greater proportion of females in the high inflammation bipolar disorder group compared to low inflammation controls, low inflammation schizophrenia and high inflammation schizophrenia (Pearson´s Chi-Square test, χ^2^(4)=10.62, all *p*<0.05). There was a significantly greater proportion of suicides in the low inflammation schizophrenia and bipolar disorder groups than in all other groups (all *p*<0.05). RIN correlated with brain pH (rho=0.31, *p*=0.002), but age, PMI, brain pH and RIN did not significantly correlate with each other (all *p*>0.085).

**Genes not fitting Gaussian distribution were log transformed**

The following gene expression data did not fit the Gaussian distribution within diagnostic groups and were log transformed: *IL6, IL6R, IL1R1, IL1B, CXCL8, IL6ST, SERPINA3, CD14, FCGR3A* and *ICAM1.* The following genes did not fit the Gaussian distribution within the inflammatory subgroups and were log transformed: *IL6, IL1R1, IL1B, CXCL8, SERPINA3, CD163, FCGR3A* and *ICAM1*.

**Relationships between demographic variables and target gene expression**

We assessed the relationships between age, PMI, RIN and brain pH and target gene expression with Pearson´s product-moment or Spearman’s rank correlations (**Supplementary Table S2**). Age negatively correlated with *pan-GFAP* and *P2RY12* mRNAs. RIN negatively correlated with *IL6*, *IL6R*, *IL1R1* and *CD14* mRNAs but positively correlated with *IBA1, P2RY12* and *P2RY13* mRNAs. PMI negatively correlated with *IL6ST, P2RY12, SERPINA3* and *VIM* mRNAs. Brain pH negatively correlated with *IL6*, *IL6R*, *IL1B*, *IL1R1*, *CXCL8*, *IL6ST* and *FCGR3A* mRNAs, and positively correlated with *P2RY12* and *P2RY13* mRNAs.

**Analysis of sex and hemisphere on target gene expression**

There was a main effect of sex on *IL1R1* expression, whereby females had higher mRNA expression compared to males [ANCOVA, *F*(2,87)=4.89, *p*=0.03]. There were no other significant main effects of sex on any other target genes. There was a main effect of hemisphere on *IL6ST* expression, whereby the right hemispheres had higher mRNA expression compared to the left hemispheres [ANCOVA, *F*(2,87)=6.64, *p*=0.01]. There were no significant interactions between sex and diagnosis or hemisphere and diagnosis (*p*≥0.05).

**Analysis of clinical characteristics across inflammatory subgroups**

The majority of schizophrenia cases were undifferentiated (**Supplementary Table S5**), and the proportion of undifferentiated cases did not significantly differ by inflammatory subgroups. The majority of bipolar disorder cases were BP-I as opposed to BP-II (**Supplementary Table S5**), and the proportion of BP-I or BP-II did not differ by inflammatory subgroups. There was a greater proportion of bipolar disorder cases with psychosis in the high (78%) compared to the low inflammation bipolar disorder subgroup (42%); however, this proportion was not different statistically (Fisher’s exact test, *p*=0.08). While there were more suicides in the low compared to high inflammation subgroup of schizophrenia (**Supplementary Table S5**), this difference did not reach statistical significance (Fisher’s exact test, *p*=0.06). The low inflammation bipolar disorder subgroup (58%) had a larger proportion of death by suicide than the high inflammation subgroup (11%, Fisher’s exact test, *p*=0.04).

A review of the clinical records demonstrated that 75 individuals had information available regarding evidence of peripheral inflammation (**Supplementary Table S5**). In the low inflammation subgroup, 48% of unaffected controls, 63% of cases with schizophrenia and 32% of cases with bipolar disorder had shown evidence of peripheral inflammation. In the high inflammation subgroup, 67% of unaffected controls, 100% of cases with schizophrenia and 78% of cases with bipolar disorder had shown evidence of peripheral inflammation. In the entire cohort, we identified a significant association between individuals in the high inflammation subgroup and evidence of peripheral inflammation [no (low inflammation subgroup/ high inflammation subgroup): 23/1, yes (low inflammation subgroup/ high inflammation subgroup): 31/20, Fisher’s exact test, *p*=0.002]. When analysing diagnostic groups separately, we further identified a significant association between individuals in the high inflammation subgroup and evidence of peripheral inflammation in bipolar disorder [no (low inflammation subgroup/ high inflammation subgroup): 12/1, yes (low inflammation subgroup/ high inflammation subgroup): 6/7, Fisher’s exact test, *p*=0.03], while the association showed a trend towards significance in schizophrenia [no (low inflammation subgroup/ high inflammation subgroup): 6/0, yes (low inflammation subgroup/ high inflammation subgroup): 12/11, Fisher’s exact test, *p*=0.06]. This analysis was not performed for the control group due to low number of cases in the high inflammation subgroup.

**
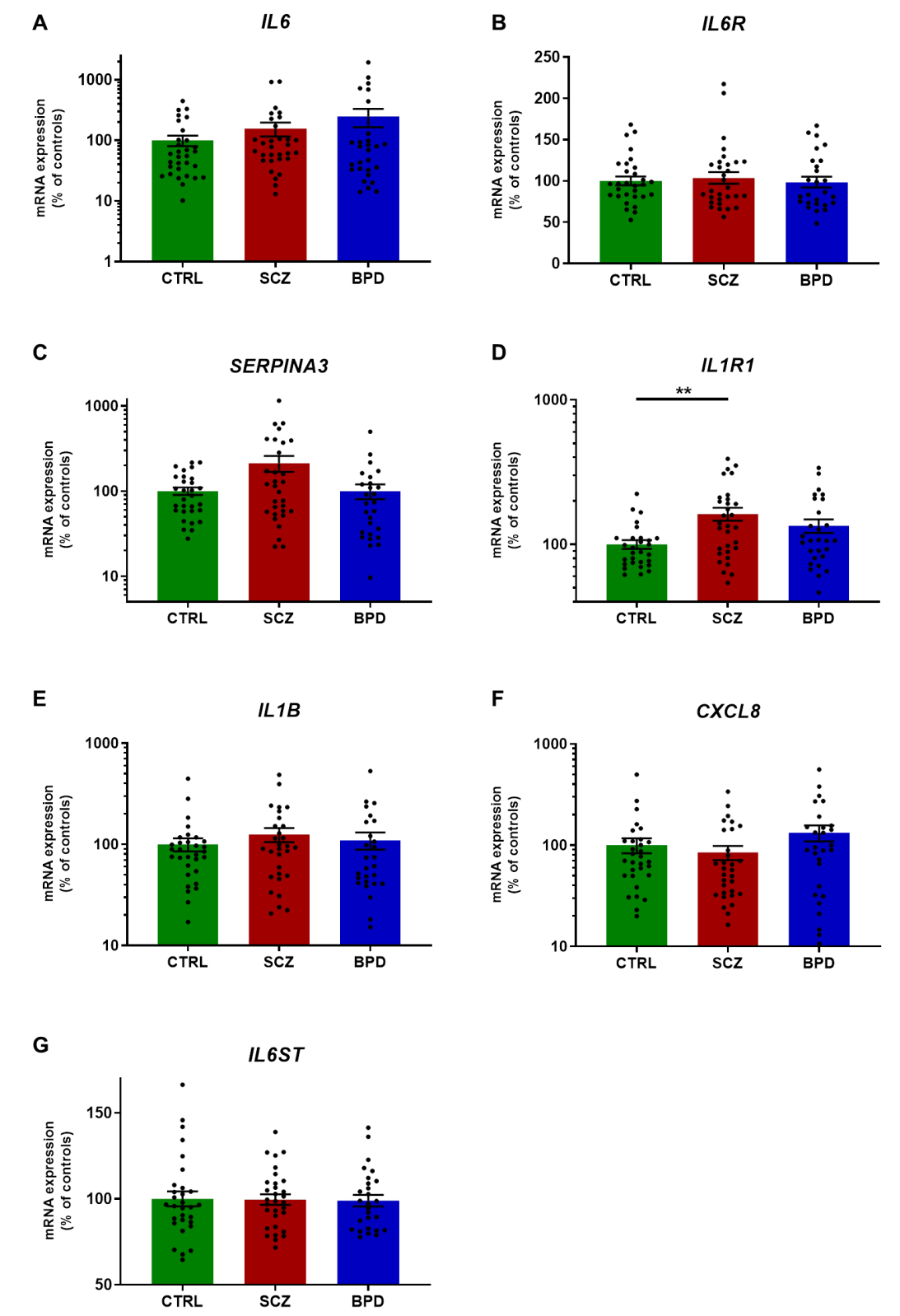
**

**Supplementary Figure S1: Inflammatory molecule gene expression in the SEZ in psychiatric conditions and unaffected controls.** *IL1R1* mRNA expression was significantly increased in schizophrenia compared to controls. No other genes significantly differed across diagnosis. Data are plotted relative to the mean of the control group (100%) ± standard error of the mean. BPD, bipolar disorder; CTRL, controls; SCZ, schizophrenia. ***p*<0.01.


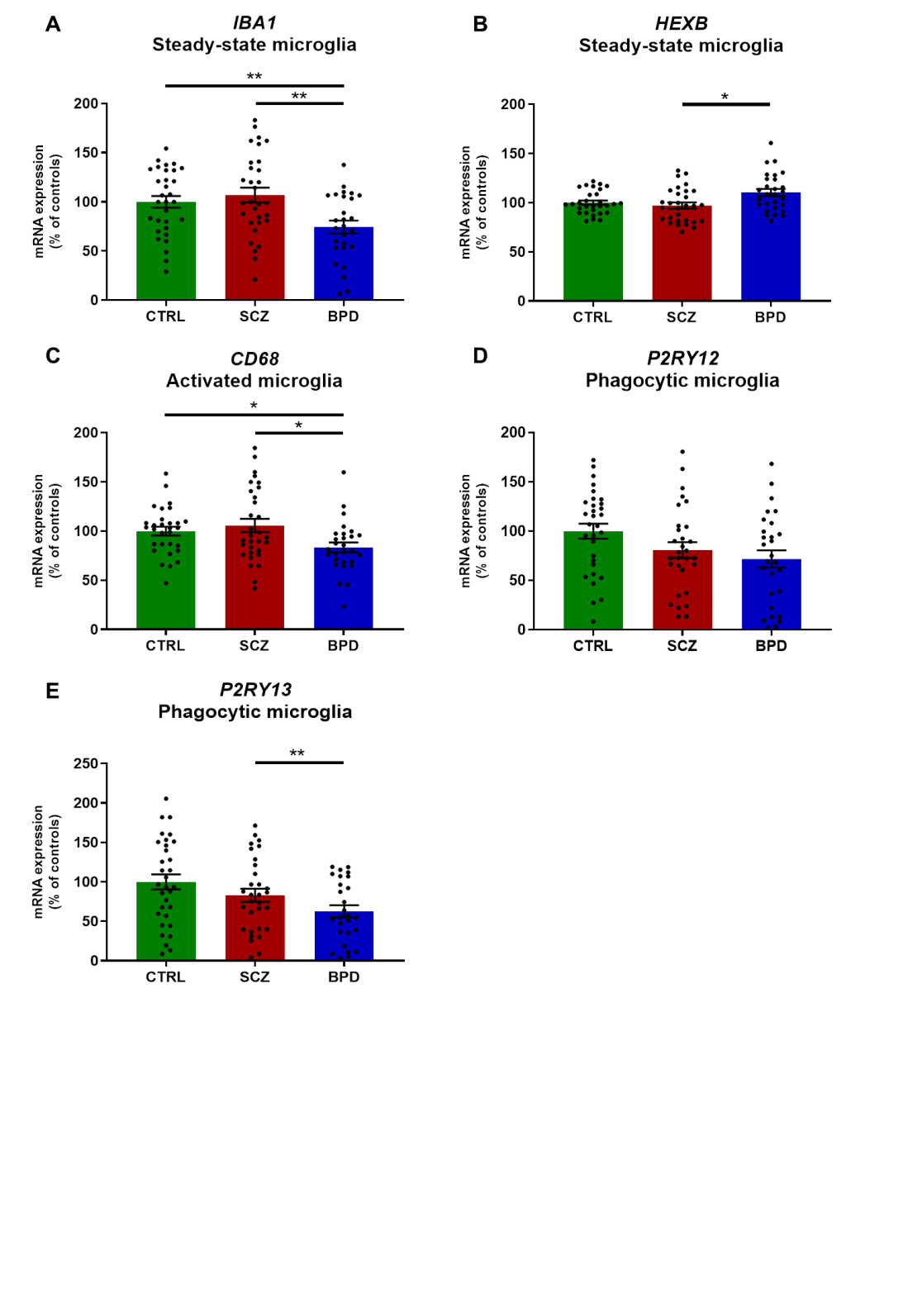


**Supplementary Figure S2: Microglia marker gene expression in the SEZ in psychiatric conditions and unaffected controls.** *IBA1* and *CD68* mRNAs were significantly reduced in bipolar disorder compared to controls and schizophrenia. *P2RY13* mRNA was significantly reduced and *HEXB* mRNA was significantly increased in bipolar disorder compared to schizophrenia. No other microglia markers significantly differed by diagnosis. Data are plotted relative to the mean of the control group (100%) ± standard error of the mean. BPD, bipolar disorder; CTRL, controls; SCZ, schizophrenia. **p*<0.05, ***p*<0.01.


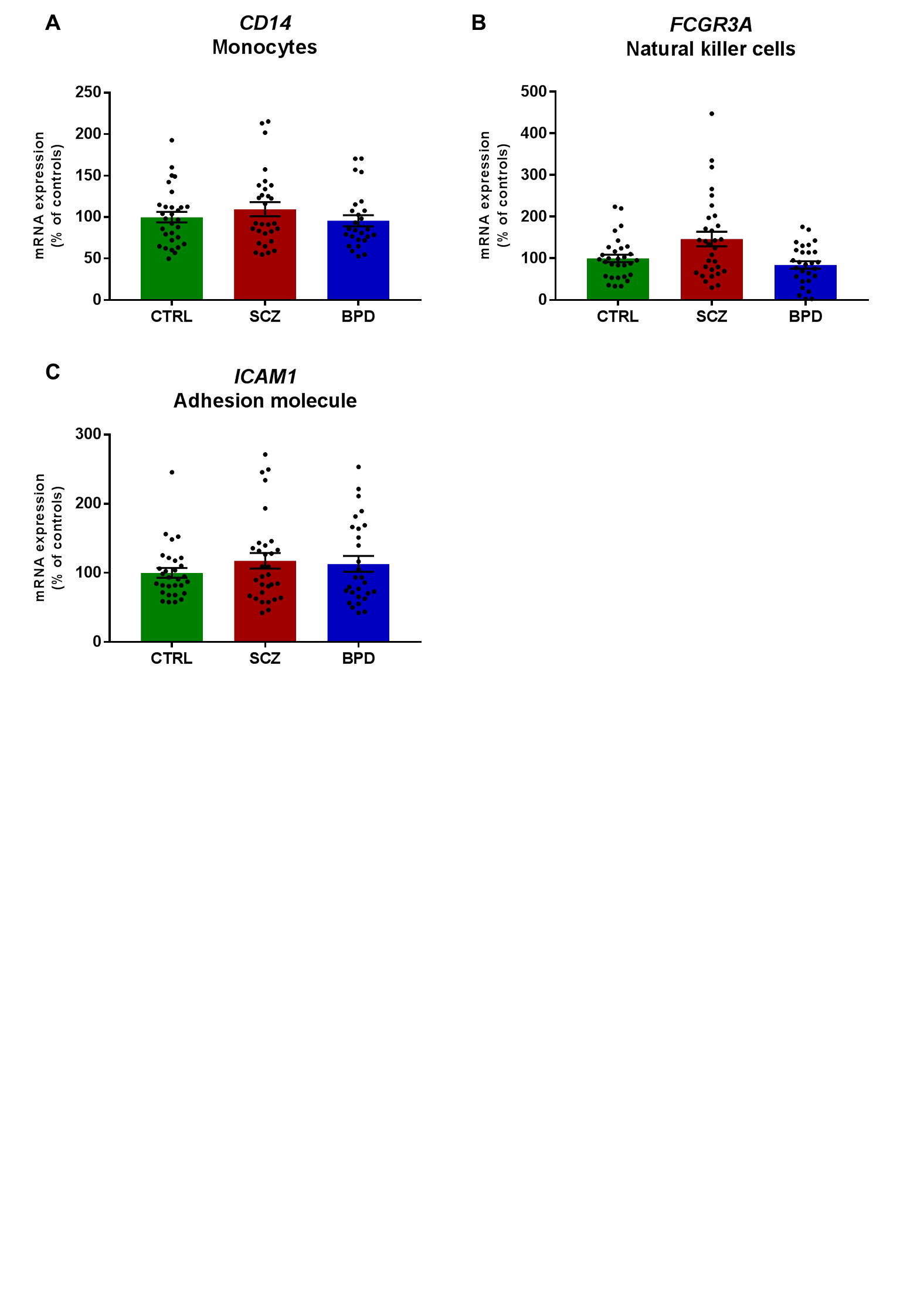


**Supplementary Figure S3: Immune cell marker gene expression in the SEZ in psychiatric conditions and unaffected controls.** *CD14*, *FCGR3A* and *ICAM1* mRNA expression did not differ across diagnosis. Note that *CD163*, *CD64* and neurogenesis markers were previously analysed by diagnosis.^1^ Data are plotted relative to the mean of the control group (100%) ± standard error of the mean. BPD, bipolar disorder; CTRL, controls; SCZ, schizophrenia.

**Supplementary References**

1. Weissleder C, North HF, Bitar M, Fullerton JM, Sager R *et al*. Reduced adult neurogenesis is associated with increased macrophages in the subependymal zone in schizophrenia. *Mol Psychiatry* 2021. doi: 10.1038/s41380-021-01149-3. Online ahead of print.
