# Supplementary material for: A schizophrenia subgroup with elevated inflammation displays reduced microglia, increased peripheral immune cell and altered neurogenesis marker gene expression in the subependymal zone
Source: Transl Psychiatry. 2021 Dec 15;11:635. doi: 10.1038/s41398-021-01742-8 (PMC8674325; doi:10.1038/s41398-021-01742-8)
Supplement: Supplementary file 2 — Supplementary Tables [file 41398_2021_1742_MOESM2_ESM.docx]

**Supplementary Table S1**: TaqMan gene expression probes

| Gene name | Gene symbol | Taqman assay ID |
| --- | --- | --- |
| Achaete-scute homolog 1 | *ASCL1* | Hs00269932_m1 |
| Antigen KI-67 | *MKI67* | Hs01032443_m1 |
| CD68 molecule | *CD68* | Hs00154355_m1 |
| Cluster of differentiation 14 | *CD14* | Hs02621496_s1 |
| Cluster of differentiation 163 | *CD163* | Hs00174705_m1 |
| Distal-less homeobox 6 antisense RNA 1 | *DLX6-AS1* | Hs03680408_m1 |
| Doublecortin | *DCX* | Hs01035496_m1 |
| Fc fragment of IgG receptor Ia | *CD64* | Hs00174081_m1 |
| Glial fibrillary acidic protein delta | *GFAPD* | AILJJYD |
| Glyceraldehyde 3-phosphate dehydrogenase | *GAPDH* | Hs99999905_m1 |
| Hexosaminidase subunit beta | *HEXB* | Hs01077594_m1 |
| Intercellular adhesion molecule 1 | *ICAM1* | Hs00164932_m1 |
| Interleukin 1 beta | *IL1B* | Hs01555410_m1 |
| Interleukin 1 receptor 1 | *IL1R1* | Hs00991002_m1 |
| Interleukin 6 | *IL6* | Hs00174131_m1 |
| Interleukin 6 receptor | *IL6R* | Hs01075664_m1 |
| Interleukin 6 signal transducer | *IL6ST* | Hs01006741_m1 |
| Interleukin 8 | *CXCL8* | Hs00174103_m1 |
| Ionized calcium-binding adapter molecule 1 | *IBA1* | Hs00741549_g1 |
| Low affinity immunoglobulin gamma Fc region receptor III-A | *FCGR3A* | Hs02388314_m1 |
| Pan glial fibrillary acidic protein | *pan-GFAP* | Hs00909236_m1 |
| Purinergic receptor P2Y12 | *P2RY12* | Hs00224470_m1 |
| Purinergic receptor P2Y13 | *P2RY13* | Hs01090437_g1 |
| Ubiquitin C | *UBC* | Hs00824723_m1 |
| Serpin family A member 3 | *SERPINA3* | Hs00153674_m1 |
| TATA-box binding protein | *TBP* | Hs00427620_m1 |
| Vimentin | *VIM* | Hs00958111_m1 |

**Supplementary Table S2**: Relationships between demographic variables and gene expression

|  | Age | | PMI | | Brain pH | | RIN | |
| --- | --- | --- | --- | --- | --- | --- | --- | --- |
|  | r/rho | *p* | r/rho | *p* | r/rho | *p* | r/rho | *P* |
| *CD14* | -0.120 | 0.253 | -0.171 | 0.101 | -0.175 | 0.094 | **-0.239** | **0.021** |
| *CD68* | -0.092 | 0.394 | -0.181 | 0.089 | -0.060 | 0.579 | 0.149 | 0.162 |
| *CXCL8* | -0.183 | 0.084 | 0.199 | 0.061 | **-0.225** | **0.033** | -0.088 | 0.407 |
| *FCGR3A* | -0.076 | 0.478 | -0.203 | 0.055 | **-0.215** | **0.042** | 0.086 | 0.422 |
| *HEXB* | 0.204 | 0.057 | 0.047 | 0.667 | -0.030 | 0.782 | -0.006 | 0.955 |
| *IBA1* | -0.036 | 0.739 | -0.144 | 0.180 | 0.102 | 0.342 | **0.444** | **<0.001** |
| *ICAM1* | -0.088 | 0.402 | -0.174 | 0.095 | -0.170 | 0.102 | -0.180 | 0.085 |
| *IL1B* | -0.069 | 0.525 | -0.166 | 0.123 | **-0.394** | **<0.001** | 0.028 | 0.796 |
| *IL1R1* | -0.027 | 0.806 | -0.161 | 0.134 | **-0.263** | **0.013** | **-0.246** | **0.021** |
| *IL6* | -0.095 | 0.374 | 0.025 | 0.814 | **-0.422** | **<0.001** | **-0.302** | **0.004** |
| *IL6R* | -0.063 | 0.564 | -0.185 | 0.088 | **-0.410** | **<0.001** | **-0.302** | **0.005** |
| *IL6ST* | -0.039 | 0.719 | **-0.299** | **0.005** | **-0.286** | **0.007** | -0.059 | 0.583 |
| *pan-GFAP* | **-0.225** | **0.036** | -0.069 | 0.524 | -0.077 | 0.481 | -0.010 | 0.926 |
| *P2RY12* | **-0.217** | **0.041** | **-0.211** | **0.047** | **0.225** | **0.034** | **0.565** | **<0.001** |
| *P2RY13* | -0.049 | 0.649 | -0.188 | 0.075 | **0.267** | **0.011** | **0.517** | **<0.001** |
| *SERPINA3* | -0.131 | 0.224 | **-0.212** | **0.048** | -0.192 | 0.073 | -0.124 | 0.250 |
| *VIM* | 0.021 | 0.846 | **-0.239** | **0.026** | -0.123 | 0.255 | -0.121 | 0.264 |

r, Pearson's correlation coefficient; rho, Spearman’s rank correlation coefficient. Bold type = *p*≤0.05.

**Supplementary Table S3**: Relationships between target gene expression and clinical variables in schizophrenia and bipolar disorder

|  | **Schizophrenia** | | | | | | **Bipolar disorder** | | | | | |
| --- | --- | --- | --- | --- | --- | --- | --- | --- | --- | --- | --- | --- |
|  | Fluphenazine equivalents | | Age of onset | | Duration of illness | | Fluphenazine equivalents | | Age of onset | | Duration of illness | |
|  |  |  |  |  |  |  |  |  |  |  |  |  |
|  | rho | *p* | rho | *p* | rho | *p* | rho | *p* | rho | *P* | rho | *p* |
| *CD14* | 0.233 | 0.200 | **-0.356** | **0.046** | 0.249 | 0.170 | -0.151 | 0.536 | **-0.431** | **0.020** | 0.094 | 0.627 |
| *CD68* | 0.243 | 0.188 | -0.272 | 0.138 | 0.255 | 0.166 | -0.097 | 0.701 | -0.283 | 0.144 | -0.098 | 0.619 |
| *CXCL8* | 0.073 | 0.698 | -0.142 | 0.447 | 0.016 | 0.932 | 0.212 | 0.397 | 0.066 | 0.740 | **-0.392** | **0.039** |
| *FCGR3A* | 0.329 | 0.071 | -0.260 | 0.158 | 0.246 | 0.182 | -0.079 | 0.747 | -0.046 | 0.819 | -0.306 | 0.120 |
| *HEXB* | 0.102 | 0.590 | 0.141 | 0.456 | 0.149 | 0.433 | 0.240 | 0.322 | 0.045 | 0.819 | 0.070 | 0.724 |
| *IBA1* | 0.116 | 0.535 | -0.160 | 0.389 | 0.204 | 0.272 | -0.219 | 0.398 | -0.240 | 0.228 | 0.084 | 0.676 |
| *ICAM1* | 0.332 | 0.068 | -0.230 | 0.214 | 0.145 | 0.436 | 0.008 | 0.974 | -0.372 | 0.051 | -0.138 | 0.483 |
| *IL1B* | **0.482** | **0.007** | 0.003 | 0.987 | 0.189 | 0.318 | -0.108 | 0.670 | **-0.455** | **0.017** | 0.139 | 0.488 |
| *IL1R1* | 0.273 | 0.137 | **-0.387** | **0.031** | 0.337 | 0.064 | 0.463 | 0.053 | 0.052 | 0.791 | 0.032 | 0.871 |
| *IL6* | 0.289 | 0.115 | -0.350 | 0.053 | **0.366** | **0.043** | 0.371 | 0.129 | 0.028 | 0.889 | -0.104 | 0.597 |
| *IL6R* | 0.229 | 0.224 | **-0.380** | **0.038** | **0.365** | **0.047** | -0.022 | 0.932 | -0.075 | 0.706 | -0.061 | 0.759 |
| *IL6ST* | -0.146 | 0.442 | 0.017 | 0.930 | 0.056 | 0.767 | 0.039 | 0.129 | -0.159 | 0.428 | -0.012 | 0.952 |
| *pan-GFAP* | -0.212 | 0.270 | **-0.406** | **0.029** | 0.150 | 0.436 | -0.295 | 0.250 | **-0.427** | **0.026** | -0.108 | 0.591 |
| *P2RY12* | **-0.417** | **0.022** | 0.352 | 0.056 | **-0.388** | **0.034** | -0.236 | 0.345 | -0.354 | 0.065 | 0.006 | 0.976 |
| *P2RY13* | -0.250 | 0.176 | 0.277 | 0.132 | -0.259 | 0.160 | -0.192 | 0.460 | -0.341 | 0.082 | 0.090 | 0.657 |
| *SERPINA3* | **0.442** | **0.014** | **-0.385** | **0.036** | **0.455** | **0.012** | 0.144 | 0.581 | **-0.422** | **0.028** | -0.157 | 0.435 |
| *VIM* | 0.105 | 0.581 | **-0.408** | **0.025** | 0.360 | 0.051 | -0.127 | 0.628 | **-0.413** | **0.032** | -0.021 | 0.918 |

rho, Spearman’s rank correlation coefficient. Bold type = *p*≤0.05.

**Supplementary Table S4**: Relationships between target gene expression and history of antidepressant use

|  | Antidepressant use | |
| --- | --- | --- |
|  | **Schizophrenia** | **Bipolar disorder** |
|  | *p* | *p* |
| *CD14* | 0.219 | 0.654 |
| *CD68* | 0.341 | 0.564 |
| *CXCL8* | 0.184 | 0.082 |
| *FCGR3A* | **0.028** | **0.027** |
| *HEXB* | **0.037** | **0.006** |
| *IBA1* | 0.464 | **0.044** |
| *ICAM1* | 0.321 | 0.276 |
| *IL1B* | 0.219 | 0.264 |
| *IL1R1* | 0.848 | 0.286 |
| *IL6* | 0.654 | 0.853 |
| *IL6R* | 0.277 | 0.944 |
| *IL6ST* | 0.422 | 0.473 |
| *pan-GFAP* | 0.866 | 0.060 |
| *P2RY12* | 0.203 | **0.011** |
| *P2RY13* | 0.418 | 0.093 |
| *SERPINA3* | 0.909 | 0.286 |
| *VIM* | 0.922 | 0.209 |

*p* values from *t*-tests or Mann-Whitney *U* tests. Bold type = *p*≤0.05.

**Supplementary Table S5**: Demographic variables of post-mortem cohort based on inflammatory subgroups

|  | **Low inflammation controls** | **Low inflammation schizophrenia** | **High inflammation schizophrenia** | **Low inflammation bipolar disorder** | **High inflammation bipolar disorder** |
| --- | --- | --- | --- | --- | --- |
| n | 27 | 19 | 11 | 19 | 9 |
| Age at death in years (range) | 45.8±6.6 (32-60) | 40.84±9.28 (19-54) | 45.72±7.02 (35-59) | 44.95±12.90 (19-63) | 46.33±8.19 (35-64) |
| pH (range) | 6.67±0.24 (6.00-7.03) | 6.5±0.23 (6.1-6.8) | 6.44±0.29 (5.9-6.93) | 6.55±0.26 (5.97-6.97) | 6.34±0.22 (5.92-6.65) |
| PMI in hours (range) | 30.18±11.81 (9-58) | 29.05±14.01 (9-65) | 33.73±18.98 (9-80) | 40.74±19.75 (12-84) | 39.44±19.29 (16-77) |
| RIN (range) | 8.03±0.44 (7.10-8.80) | 7.8±0.83 (6.10-9.10) | 7.53±0.89 (5.10-8.50) | 7.99± 0.60 (6.20-8.70) | 7.22±0.91 (6.00-8.40) |
| Sex | 21M/ 6F | 14M/ 5F | 8M/ 3F | 11M/ 8F | 2M/ 7F |
| Hemisphere | 15L/ 12R | 9L/ 10R | 5L/ 6R | 12L/ 7R | 6L/ 3R |
| Age of onset in years | NA | 22.42±6.93 | 18.45±3.01 | 25.11±10.25 | 23.89±7.18 |
| Duration of illness in years | NA | 18.42±10.84 | 27.18±7.15 | 19.84±10.08 | 22.44±10.01 |
| Lifetime antipsychotic dose (fluphenazine equivalents in mg) | NA | 57376±95382 | 125909±92596 | 7654±7546 | 31571±44843 |
| Psychotic feature^a^ | yes=0, no=27 | yes=19, no=0 | yes=11, no=0 | yes=8, no=10, unknown=1 | yes=7, no=1, unknown=1 |
| Antidepressant use^b^ | yes=0, no=27 | yes=6, no=13 | yes=2, no=9 | yes=11, no=8 | yes=7, no=2 |
| Types of antidepressants | NA | SSRI=4 (fluoxetine=1), TCA=1, others=1 | SARI=1, SSRI=1 (fluoxetine=1) | SARI=4, SNRI=1, SSRI=6 (fluoxetine=3), TCA=2, others=2 | SARI=3, SNRI=3, SSRI=3 (fluoxetine=2), TCA=2 |
| Smoking at time of death^a^ | yes=9, no=7, unknown=11 | yes=10, no=4, unknown=5 | yes=9, no=0, unknown=2 | yes=7, no=5, unknown=7 | yes=6, no=1, unknown=2 |
| Manner of death | natural=27, suicide=0 | natural=13, suicide=6 | natural=11, suicide=0 | natural=8, suicide=11 | natural=8, suicide=1 |
| Evidence of peripheral inflammation^a^ | yes=13, no=5, unknown=9 | yes=12, no=6, unknown=1 | yes=11, no=0, unknown=0 | yes=6, no=12, unknown=1 | yes=7, no=1, unknown=1 |
| Diagnostic subtypes | NA | disorganised=1, paranoid=3, undifferentiated=15 | disorganised=0, paranoid=3, undifferentiated=8 | BP-I=14, BP-II=4, BP not otherwise specified=1, BP not otherwise specified possible psychosis=0, BP type- schizoaffective=0 | BP-I=7, BP-II=0, BP not otherwise specified=0, BP not otherwise specified possible psychosis=0, BP type- schizoaffective=1 |
| Lifetime alcohol use | little or none=12, social=10, moderate past=1, moderate present=2, heavy past=2, heavy present=0 | little or none=8, social=4, moderate past=0, moderate present=0, heavy past=1, heavy present=6 | little or none=2, social=3, moderate past=2, moderate present=2, heavy past=1, heavy present=1 | little or none=1, social=4, moderate past=4, moderate present=3, heavy past=1, heavy present=6 | little or none=2, social=2, moderate past=1, moderate present=0, heavy past=3, heavy present=1 |
| Lifetime drug use | little or none=24, social=2, moderate past=0, moderate present=1, heavy past=0, heavy present=0 | little or none=10, social=1, moderate past=3, moderate present=0, heavy past=1, heavy present=4 | little or none=4, social=1, moderate past=0, moderate present=3, heavy past=0, heavy present=1, unknown=2 | little or none=5, social=2, moderate past=5, moderate present=1, heavy past=2, heavy present=4 | little or none=3, social=1, moderate past=1, moderate present=2, heavy past=2, heavy present=0 |

Data are shown as mean ± standard deviation. Ranges are presented in parentheses. BP, bipolar disorder; F, female; L, left; M, male; PMI, post-mortem interval; R, right; RIN, RNA integrity number; SARI, serotonin antagonist and reuptake inhibitor; SSRI, selective serotonin reuptake inhibitor; SNRI, serotonin-norepinephrine reuptake inhibitor; TCA, tricyclic antidepressant. ^a^Unknown individuals were excluded from statistical analysis. ^b^Some cases were prescribed multiple antidepressant medications.
